# Anti-α-Glucosidase, SAR Analysis, and Mechanism Investigation of Indolo[1,2-b]isoquinoline Derivatives

**DOI:** 10.3390/molecules28135282

**Published:** 2023-07-07

**Authors:** Mengyue Li, Lin Li, Li Lu, Xuetao Xu, Jinhui Hu, Jin-Bao Peng

**Affiliations:** School of Biotechnology and Health Sciences, Wuyi University, Jiangmen 529020, China

**Keywords:** indolo[1,2-b]isoquinoline, SAR analysis anti-α-glucosidase, mechanism

## Abstract

To find potential α-glucosidase inhibitors, indolo[1,2-b]isoquinoline derivatives (**1**–**20**) were screened for their α-glucosidase inhibitory effects. All derivatives presented potential α-glucosidase inhibitory effects with IC_50_ values of 3.44 ± 0.36~41.24 ± 0.26 μM compared to the positive control acarbose (IC_50_ value: 640.57 ± 5.13 μM). In particular, compound **11** displayed the strongest anti-α-glucosidase activity, being ~186 times stronger than acarbose. Kinetic studies found that compounds **9**, **11**, **13**, **18**, and **19** were all reversible mix-type inhibitors. The 3D fluorescence spectra and CD spectra results revealed that the interaction between compounds **9**, **11**, **13**, **18**, and **19** and α-glucosidase changed the conformational changes of α-glucosidase. Molecular docking and molecular dynamics simulation results indicated the interaction between compounds and α-glucosidase. In addition, cell cytotoxicity and drug-like properties of compound **11** were also investigated.

## 1. Introduction

Diabetes mellitus, one of the most common endocrine and metabolic diseases, is induced by insulin resistance or abnormality [1]. Diabetes mellitus patients have abnormally elevated blood glucose, and long-term high blood glucose levels can harm vascular endothelium, which can then lead to various complications. Using hypoglycemic drugs to regulate postprandial hyperglycemia is an important strategy for type 2 diabetes patients [2,3,4].

α-Glucosidase, a crucial membrane-bound enzyme in the small intestine, is one of the most important therapeutic targets for diabetes [5,6,7]. It is responsible for the hydrolysis of glycosidic linkage bonds of carbohydrates, especially disaccharides and polysaccharides, thereby releasing absorbable monosaccharides, which are absorbed to cause postprandial hyperglycemia [8,9]. Therefore, the inhibition of α-glucosidase activity can delay carbohydrate ingestion and reduce postprandial hyperglycemia [10]. Up to now, increasingly more α-glucosidase inhibitors have been developed, but only a few inhibitors are clinically used for the treatment of type 2 diabetes, such as acarbose, voglibose, and miglitol [11,12,13]. Moreover, long-term use of these drugs also leads to some gastrointestinal side effects, including diarrhea and flatulence [14,15]. This situation has encouraged the authors to develop more efficient and safer α-glucosidase inhibitors [16,17,18].

**Figure 1:**
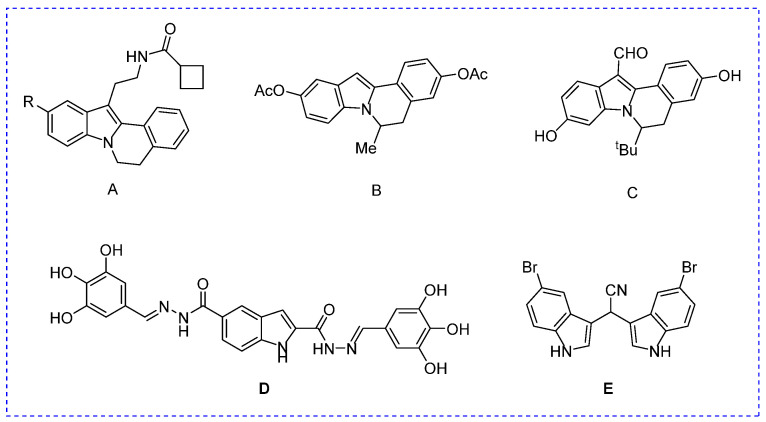
The structure of some indolo[1,2-b]isoquinolines derivatives and our previous work. (**A**) Melatonin antagonist; (**B**) Inhibitor of estrogen receptor; (**C**) Inhibitor of tubulin polymerization; (**D**) Inhibitor of α-glucosidase; (**E**) Inhibitor of α-glucosidase.

The indole and isoquinoline scaffolds are two frequent structural units of alkaloids and have attracted considerable attention due to their potent biological activities [19,20]. Specifically, many indole or isoquinoline derivatives display potential anti-α-glucosidase and anti-diabetes activities [21,22,23,24]. Indolo[1,2-b]isoquinoline is a fused structure of indole and isoquinoline rings; therefore, they exhibit partial or more comprehensive activity compared to indole and isoquinoline scaffolds [25,26,27]. The derivatives ubiquitously exist in natural products and synthetic biologically active molecules and have gained great attention due to their numerous biological activities. For example, indolo[1,2-b]isoquinoline derivatives (Figure 1A–C) have been reported as melatonin antagonists, estrogen receptor inhibitors, and tubulin polymerization inhibitors, respectively [27,28].

Recently, our group designed and synthesized some potential α-glucosidase inhibitors, including two series of indole derivatives (Figure 1D,E) [29,30]. In order to find indolo[1,2-b]isoquinolines with potential pharmaceutical activity, we developed an efficient synthesis strategy and obtained a series of derivatives (**1**–**20**) [25]. In consideration of these, we evaluated the anti-α-glucosidase activity of indolo[1,2-b]isoquinoline derivatives (**1**–**20**), conducted SAR analysis, revealed the inhibition mechanism, and analyzed the drug-like properties and cytotoxicity.

## 2. Results

### 2.1. Chemistry

**Figure 2:**
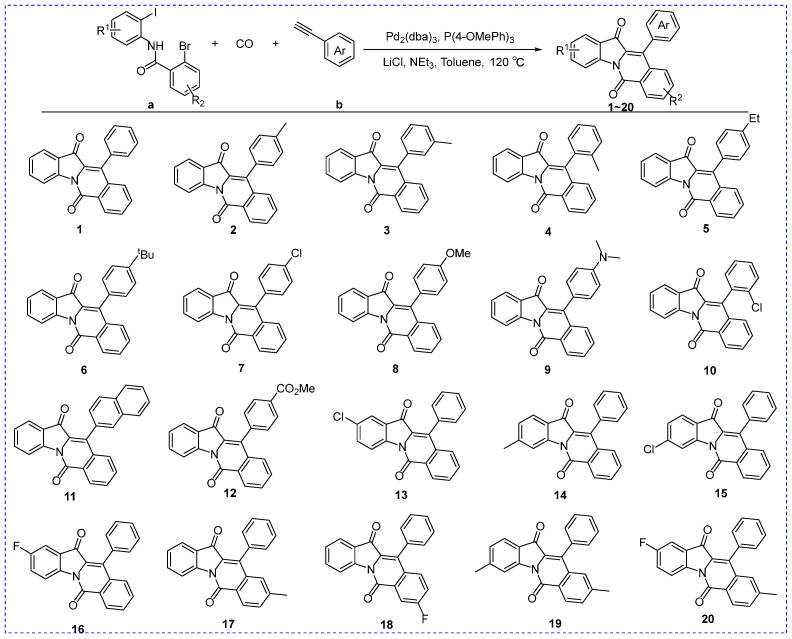
Synthesis scheme (**a**,**b**) and structure of indolo[1,2-b]isoquinoline derivatives (**1**–**20**).

All indolo[1,2-b]isoquinoline derivatives (**1**–**20**) were obtained from our previous work [25]. Their chemical synthesis routes and chemical structures are shown in Figure 2. All derivatives (**1**–**20**) could be efficiently synthesized and the NMR data were shown in Supplementary Materials.

### 2.2. α-Glucosidase Activity Evaluation

Because commercial human α-glucosidase is still lacking, the anti-α-glucosidase activity of indolo[1,2-b]isoquinoline derivatives (**1**–**20**) was first evaluated using α-glucosidase from *Saccharomyces cerevisiae*, due to its similar active region structure to human α-glucosidase. Acarbose was selected as the positive control. The 50% inhibition concentration (IC_50_) of all derivatives (**1**–**20**) was obtained and is illustrated in Table 1. The results showed that all compounds (**1**–**20**) presented good inhibitory activity against α-glucosidase with IC_50_ values ranging from 3.44 ± 0.36 to 41.24 ± 0.26 μM. However, positive control acarbose only showed an inhibitory IC_50_ value of 640.57 ± 1.13 μM, far lower than that of compounds (**1**–**20**). Furthermore, compound **11** had the strongest α-glucosidase inhibitory activity (IC_50_ = 3.44 ± 0.36 μM). Therefore, indolo[1,2-b]isoquinoline derivatives (**1**–**20**), especially compound **11**, could be used as lead compounds to develop new α-glucosidase inhibitors. Up to now, although indolo[1,2-b]isoquinolines have not been reported as α-glucosidase inhibitors, lots of indole derivatives and quinoline derivatives had been found as α-glucosidase inhibitors. Hybridization has been considered as one of the effective strategies in the development of drugs. Therefore, the good α-glucosidase inhibitory of indolo[1,2-b]isoquinolines might be attributed to the scaffold of indole, isoquinoline, and their hybrid.

### 2.3. SAR Analysis

**Figure 3:**
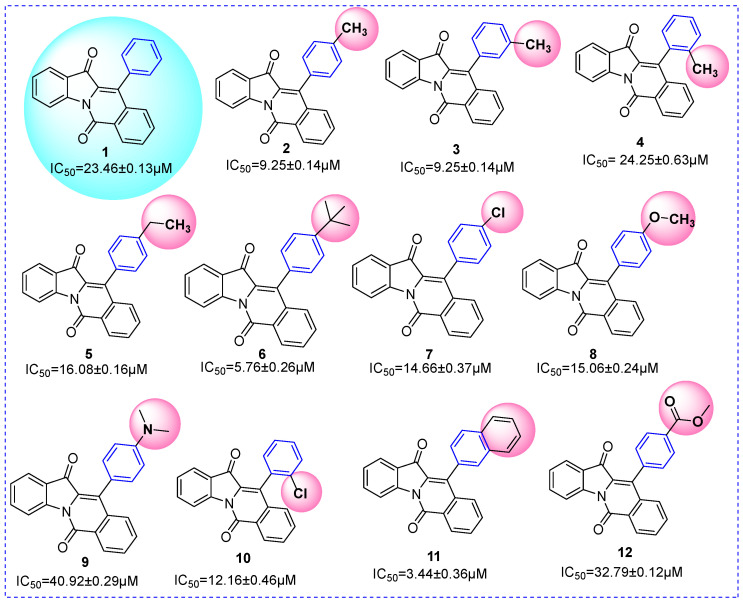
SAR analysis of compounds **1**~**12**.

In order to better guide future synthesis work, the structure-activity relationship (SAR) of compounds **1**~**20** was analyzed according to their α-glucosidase inhibitory activity (Table 1). Compound **1** (IC_50_: 23.46 ± 0.13 μM), with no substitution on the molecular skeleton, was selected as the control compound. For compounds (**2**~**12**), with different substituents at the benzene ring of R1, they all had a stronger inhibitory activity than compound **1** (Figure 3). That is, substituents (electron-donating substituent: methyl, ethyl, *t*-butyl, methoxy, naphthalene; electron-withdrawing substituent: chlorine) enhanced the inhibitory activity, while dimethylamino (electron-donating substituent) and methyl formate (electron-withdrawing substituent) reduced the inhibitory activity. For compounds (**2**~**4**) with methyl (electron-donating substituent) at different positions, 3-position methyl leads to better inhibitory activity. For compounds (**2**, **5**, **6**) with different alkyl substituents (electron-donating substituent), long-chain alkyl (*t*-butyl) was favorable to inhibitory activity.

**Figure 4:**
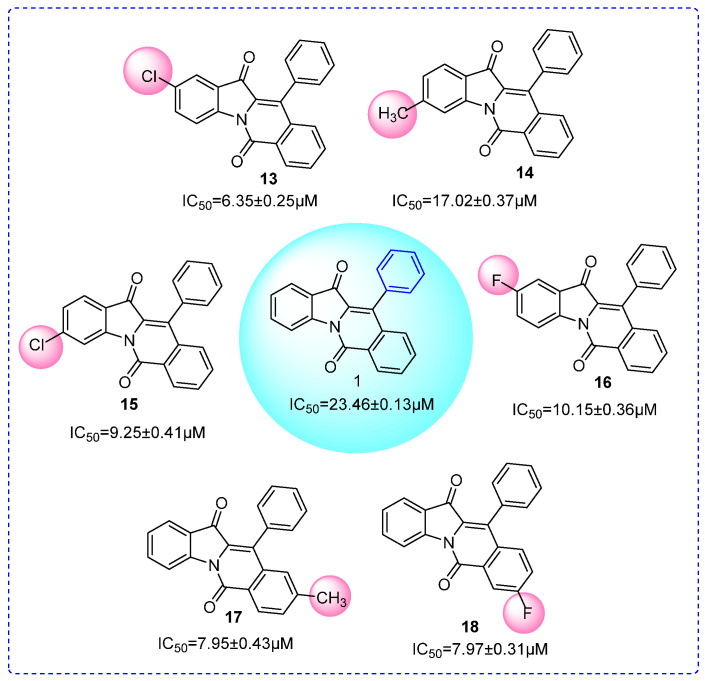
SAR analysis of compounds **1** and **13**~**18**.

For compounds (**13**~**16**), we fixed R1 as the benzene ring and changed the substituents of R2. Their inhibitory activities were also stronger than compound **1**, respectively (Figure 4), indicating that substituents (electron-donating substituent: methyl; electron-withdrawing substituent: chlorine, fluorine) could improve the inhibitory activity. For compounds (**17**~**18**), we fixed R1 as the benzene ring and changed the substituents of R3. Their inhibitory activity results indicated that substituents (electron-donating substituent: methyl; electron-withdrawing substituent: fluorine) would be helpful to the inhibitory activity (Figure 4). It could be seen from the SAR results that some electron-withdrawing substituents and electron-donating substituents would enhance the inhibitory activity, but others would reduce the inhibitory activity showing that the effects of substituents on enzyme activity were not consistent to their chemical prosperities.

### 2.4. Inhibition Kinetics Study

**Figure 5:**
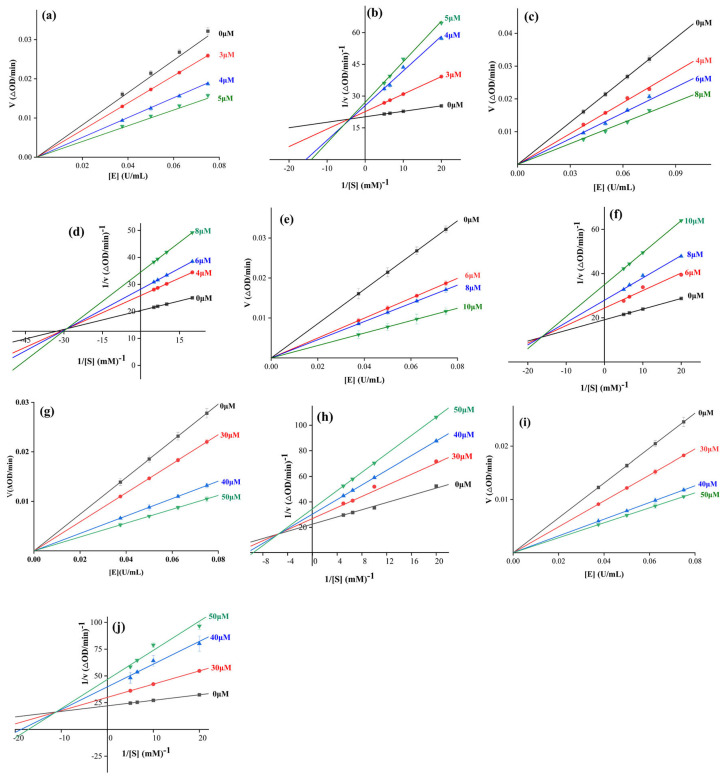
The inhibition kinetics of compounds **9**, **11**, **13**, **18**, and **19** on α-glucosidase. (**a**) Enzyme kinetics of compound **9**; (**b**) Substrate kinetics of compound **9**; (**c**) Enzyme kinetics of compound **11**; (**d**) Substrate kinetics of compound **11**; (**e**) Enzyme kinetics of compound **13**; (**f**) Substrate kinetics of compound **13**; (**g**) Enzyme kinetics of compound **18**; (**h**) Substrate kinetics of compound **18**; (**i**) Enzyme kinetics of compound **19**; (**j**) Substrate kinetics of compound **19**.

Compounds **9**, **11**, **13**, **18**, and **19** with different α-glucosidase inhibitory activity were selected as representative compounds to reveal the inhibition mechanism of indolo[1,2-b]isoquinoline derivatives (**1**–**21**) on α-glucosidase by the implementation of an inhibition kinetics study. For enzyme kinetics (Figure 5a,c,e,g,i), the plots of remaining enzyme activity versus enzyme concentration treated with compounds **9**, **11**, **13**, **18**, and **19** all passed through the origin point, which indicated that the inhibition of compounds **9**, **11**, **13**, **18**, and **19** was reversible, respectively. For substrate kinetics (Figure 5b,d,f,h,j), the Lineweaver-Burk plots of remaining enzyme activity versus enzyme concentration treated with compounds **9**, **11**, **13**, **18**, and **19** intersected at one point in the second quadrant, respectively. The inhibition kinetics study was an effective method by which to understand the inhibition mechanism of the inhibitor against the target protein. In a substrate kinetics study, different inhibition mechanisms of inhibitors manifested as different intersection positions of Lineweaver-Burk plots. Further research confirmed that the intersection position of the Lineweaver-Burk plots was located in four quadrants, meaning mixed-type inhibition [31]. Therefore, our results declared a mixed-type inhibition of compounds **9**, **11**, **13**, **18**, and **19**, respectively. Moreover, compounds **9**, **11**, **13**, **18**, and **19** bind with both free enzyme and enzyme-substrate complexes to inhibit α-glucosidase. The inhibition kinetics constants of compounds **9**, **11**, **13**, **18**, and **19** were obtained and are listed in Table 2, including *K*_i_, Kis, Km, and Vmax values, which might be helpful to understand the inhibition mechanism.

### 2.5. 3D Fluorescence Spectra Assay

**Figure 6:**
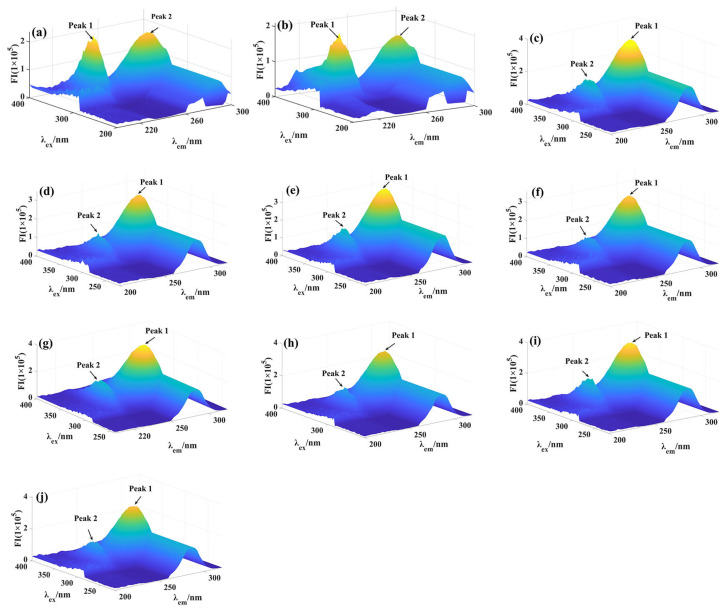
3D fluorescence spectra of α-glucosidase with compounds **9**, **11**, **13**, **18**, and **19**. (**a**) 3D fluorescent spectra of α-glucosidase; (**b**) 3D fluorescent spectra of α-glucosidase with compounds **9**; (**c**) 3D fluorescent spectra of α-glucosidase; (**d**) 3D fluorescent spectra of α-glucosidase with compounds **11**; (**e**) 3D fluorescent spectra of α-glucosidase; (**f**) 3D fluorescent spectra of α-glucosidase with compounds **13**; (**g**) 3D fluorescent spectra of α-glucosidase; (**h**) 3D fluorescent spectra of α-glucosidase with compounds **18**; (**i**) 3D fluorescent spectra of α-glucosidase; (**j**) 3D fluorescent spectra of α-glucosidase with compounds **19**.

The 3D fluorescence spectra of α-glucosidase with compounds **9**, **11**, **13**, **18**, and **19** was investigated to analyze the effect of compounds on the structure of α-glucosidase, respectively. Two important characteristic peaks appeared in the 3D fluorescence spectra of α-glucosidase (Figure 6a,c,e,g,i), including Peak **1** (λ_ex_ = 335 nm, λ_em_ = 230 nm), corresponding to the main chain structure of the polypeptide, and Peak **2** (λ_ex_ = 335 nm, λ_em_ = 277.5 nm), corresponding to tyrosine and tryptophan residues. While treatment with compounds **9**, **11**, **13**, **18**, and **19** could decrease the fluorescence intensity of Peak **1** and Peak **2**, respectively. (Figure 6b,d,f,h,j). Previously, 3D fluorescence spectra have been used to determine protein conformation changes. Our results indicated that the interaction of inhibitors with α-glucosidase changed the microenvironment and structure of α-glucosidase, consistent with previous research [32], which also indicated that the interaction of the inhibitor with α-glucosidase reduced the intensity of the 3D fluorescence spectra characteristic peaks of α-glucosidase.

### 2.6. CD Spectra Assay

**Figure 7:**
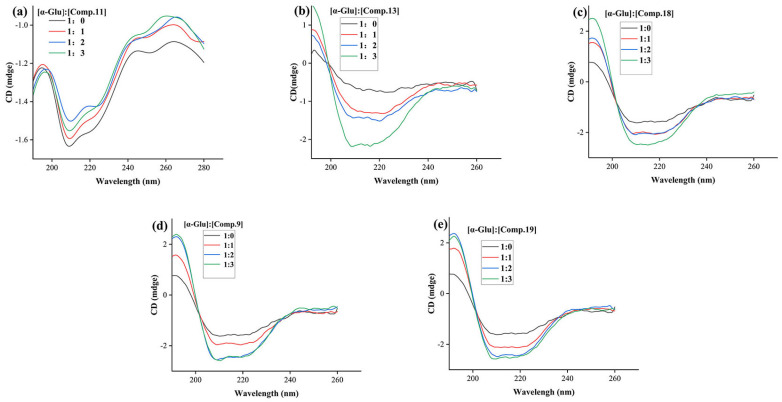
CD spectra of α-glucosidase in the presence of compounds **9**, **11**, **13**, **18**, and **19**. (**a**) CD spectra of α-glucosidase with compound **9**; (**b**) CD spectra of α-glucosidase with compound **11**; (**c**) CD spectra of α-glucosidase with compound **13**; (**d**) CD spectra of α-glucosidase with compound **18**; (**e**) CD spectra of α-glucosidase with compound **19**.

CD spectra were also monitored to study the effect of compounds **9**, **11**, **13**, **18**, and **19** on the conformational changes of α-glucosidase. As shown in Figure 7a–e, α-glucosidase presented two negative CD bands in the region of 190~280 nm, which was owed to the electronic transitions of n→π* of α-helical bonds. Treatment of compound **11** resulted in a concentration-dependent increase in the CD band intensity of α-glucosidase, while treatment of compounds **9**, **13**, **18**, and **19** led to a decrease in CD band intensity. For specific secondary structure change, treatment with compound **11** (molar ratio: 3:1) reduced α-helix (from 8.50 to 7.80%), β-sheet (from 34.10 to 35.20%), and β-turn (from 19.80 to 20.10%), and increased random coils (from 35.50 to 35.80%), respectively (Table 3). While treatment of compounds **9**, **13**, **18**, and **19** led to an increase of α-helix and a reduction of β-sheet, β-turn, and random coils, respectively (Table 3). CD spectra have been an important method by which to study protein structural changes and secondary structural content. Previous research has shown that the interaction of an inhibitor with α-glucosidase caused the partial folding and loosening of the α-glucosidase structure [33]. Our results also revealed that CD bands of α-glucosidase could be changed by the addition of compounds **9**, **11**, **13**, **18**, and **19**, suggesting an effective interaction between compounds **9**, **11**, **13**, **18**, and **19** with α-glucosidase, respectively.

### 2.7. Molecular Docking

**Figure 8:**
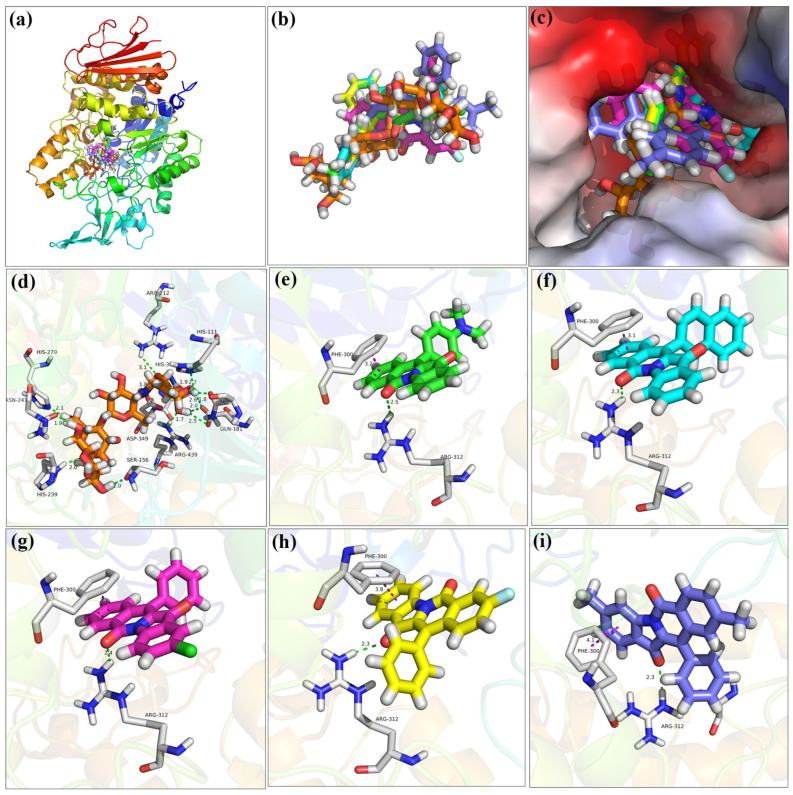
Docking of compounds **9**, **11**, **13**, **18**, **19**, and acarbose with homologous *Saccharomyces cerevisiae* α-glucosidase. (**a**) Compounds **9**, **11**, **13**, **18**, **19**, and acarbose in homologous α-glucosidase; (**b**) Compounds **9**, **11**, **13**, **18**, **19**, and acarbose; (**c**) Compounds **9**, **11**, **13**, **18**, **19**, and acarbose in pocket of homologous α-glucosidase; (**d**) Docking of acarbose with homologous α-glucosidase; (**e**) Docking of compounds **9** with homologous α-glucosidase; (**f**) Docking of compounds **11** with homologous α-glucosidase; (**g**) Docking of compounds **13** with homologous α-glucosidase; (**h**) Docking of compounds **18** with homologous α-glucosidase; (**i**) Docking of compounds **19** with homologous α-glucosidase.

Molecular docking simulation between compounds **9**, **11**, **13**, **18**, **19**, and acarbose with *Saccharomyces cerevisiae* α-glucosidase was first investigated to clear the specific binding status. As can be seen in Figure 8a–c, compounds **9**, **11**, **13**, **18**, **19**, and acarbose are all embedded in the same area of the α-glucosidase active pocket and are tightly bound to amino acid residues. For positive control acarbose (Figure 8d), there were some hydrogen bonds formed with His279 (2.1 Å), Asn241 (1.9 Å), His239 (2.0 Å), His156 (2.0 Å), Arg439 (1.7 Å), His111 (2.7 Å), Gln181 (1.8 Å), His111 (2.7 Å), His348 (3.1 Å), and Asp68 (2.0, 2.5, and 2.6 Å), respectively. As seen in Figure 8e, the docking results of compound **9**, compound **9** formed a hydrogen bond with Arg312 (2.5 Å) and one π-π bond with Phe300 (3.2 Å), respectively. Inhibitor **11** formed a hydrogen bond with Arg312 (2.3 Å) and a π-π bond with Phe177 (3.1 Å), respectively (Figure 8f). Compound **13** formed a hydrogen bond with Arg312 (2.4 Å) and one π-π bond with Phe300 (3.1 Å), respectively (Figure 8g). Compound **18** formed a hydrogen bond with Arg312 (2.3 Å) and one π-π bond with Phe300 (3.8 Å), respectively (Figure 8h). Compound **19** also formed a hydrogen bond with Arg312 (2.3 Å) and one π-π bond with Phe300 (4.1 Å), respectively (Figure 8i). The different binding interactions between compounds **9**, **11**, **13**, **18**, and **19**, with α-glucosidase would be helpful to explain the different inhibitory activites.

**Figure 9:**
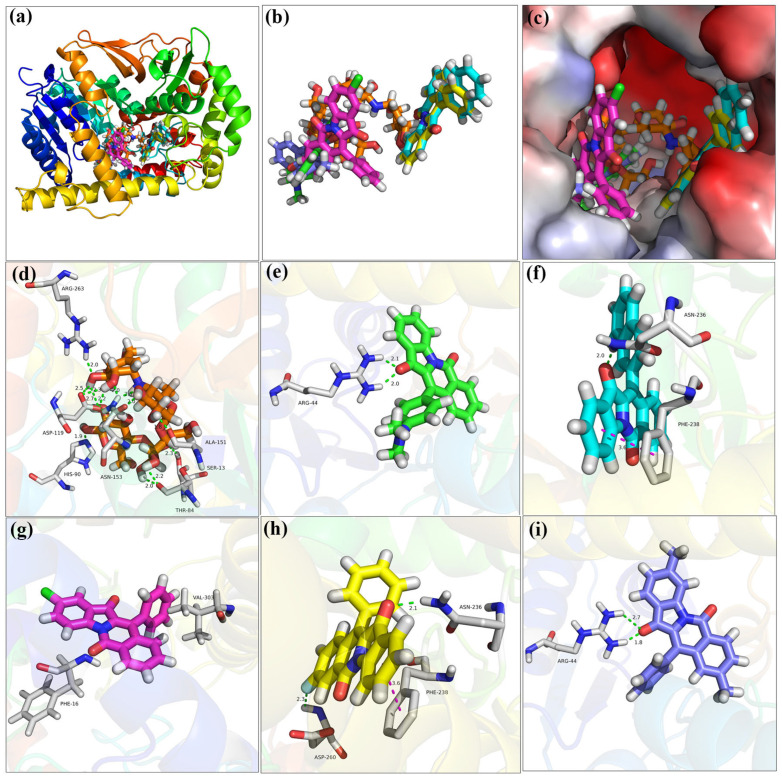
Docking of compounds **9**, **11**, **13**, **18**, **19**, and acarbose with homologous human α-glucosidase. (**a**) Compounds **9**, **11**, **13**, **18**, **19**, and acarbose in human α-glucosidase; (**b**) Compounds **9**, **11**, **13**, **18**, **19**, and acarbose; (**c**) Compounds **9**, **11**, **13**, **18**, **19**, and acarbose in pocket of human α-glucosidase; (**d**) Docking of acarbose with human α-glucosidase; (**e**) Docking of compounds **9** with human α-glucosidase; (**f**) Docking of compounds **11** with human α-glucosidase; (**g**) Docking of compounds **13** with human α-glucosidase; (**h**) Docking of compounds **18** with human α-glucosidase; (**i**) Docking of compounds **19** with human α-glucosidase.

To better validate the binding between compounds and α-glucosidase, human α-glucosidase was used as a target protein to simulate the docking; the results are shown in Figure 9. Because the 3D structure of human α-glucosidase has still not been characterized, a homologous model of human α-glucosidase was also built based on the existing enzyme. Although compounds **9**, **11**, **13**, **18**, **19**, and acarbose were embedded in the active pocket of α-glucosidase, it was observed that compounds **9**, **19**, and acarbose were located in the interior of the active pocket, and compounds **11**, **13**, and **18** were located in the exterior of the active pocket (Figure 9a–c). Acarbose made hydrogen bonds with His90 (1.9 Å), Ser13 (1.8 Å), Thr84 (2.0 and 2.2 Å), Ala151 (2.3 Å), Asn153 (2.0, 2.0 and 2.0 Å), Asp119 (2.2, 2.3, 2.5, and 2.7 Å), and Arg263 (2.0 Å), respectively (Figure 9d). Compound **9** formed two hydrogen bonds with Arg44 (2.0 and 2.1 Å) (Figure 9e). Inhibitor **11** formed a hydrogen bond with Asn236 (2.0 Å) and a π-π bond with Phe238 (3.6 Å), respectively (Figure 9f). Compound **13** only formed hydrophobic bonds (Figure 9g). Compound **18** formed two hydrogen bonds with Asn236 (2.1 Å) and Asp260 (2.3 Å), and a π-π bond with Phe238 (3.6 Å), respectively (Figure 9h). Compound **19** formed two hydrogen bonds with Arg44 (1.8 and 2.7 Å) (Figure 9i). It was observed that there were many differences between the two docking results, especially the amino acid residues in the active pocket, which formed hydrogen or π-π bonds.

### 2.8. Molecular Dynamics Simulation

**Figure 10:**
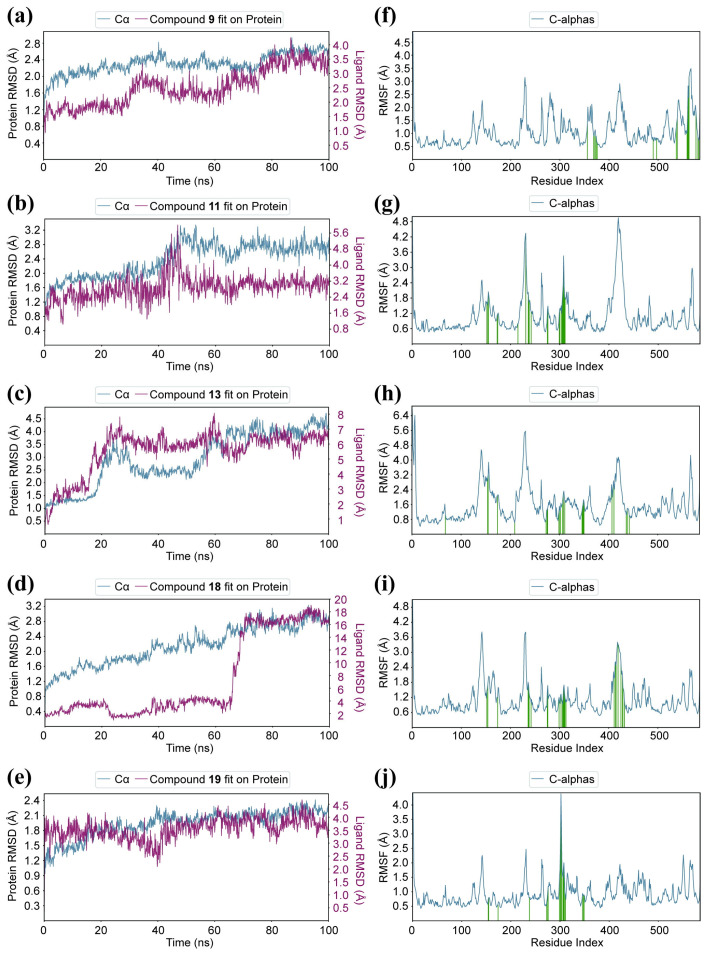
Molecular dynamics simulation of compounds **9**, **11, 13, 18**, and **19** with α-glucosidase. (**a**) RMSD of α-glucosidase with compounds **9**; (**b**) RMSF of α-glucosidase with compounds **9**; (**c**) RMSD of α-glucosidase with compounds **11**; (**d**) RMSF of α-glucosidase with compounds **11**; (**e**) RMSD of α-glucosidase with compounds **13**; (**f**) RMSF of α-glucosidase with compounds **13**; (**g**) RMSD of α-glucosidase with compounds **18**; (**h**) RMSF of α-glucosidase with compounds **18**; (**i**) RMSD of α-glucosidase with compounds **19**; (**j**) RMSF of α-glucosidase with compounds **19**.

To analyze the contribution of amino acid residues in the active pocket to substrate binding, the docking results of compounds **9**, **11**, **13**, **18**, and **19** in the complexes with α-glucosidase were further simulated using molecular dynamics simulation for 100 ns. The root-mean-square deviation (RMSD) results are presented in Figure 10a–e, illustrating the equilibration of the systems. The calculated RMSD values confirm that these systems reached a state of structural equilibrium. Specifically, it was observed that the compound **11**-α-glucosidase systems achieved stability after approximately 45 ns. The RMSD values for the free α-glucosidase and compound **11**-α-glucosidase were measured at 2.7 Å and 1.7 Å, respectively. Moreover, the overall RMSD fluctuations of the compound **11**-α-glucosidase remained within the range of 1–1.7 Å, indicating the backbone stability of α-glucosidase during the docking process. The RMSF value was also observed to characterize local changes in the protein chain (Figure 10f–j). The overall RMSF value indicated few fluctuations in the N- and C-terminal loop regions. Subsequently, the binding free energies of the complexes were calculated using the molecular mechanics−generalized Born surface area (MM-GBSA) method (Table 4). The total binding free energies were determined as follows: −47.23 kcal/mol for compound **9**, −66.94 kcal/mol for compound **11**, −60.27 kcal/mol for compound **13**, −52.87 kcal/mol for compound **18**, and −51.49 kcal/mol for compound **19** in their respective complexes with α-glucosidase. The results obtained from the molecular dynamics simulations are in agreement with the experimental observations. Notably, the contributions that favored ligand binding included van der Waals energy, electrostatic interaction energy, and nonpolar solvation interaction. Conversely, the polar solvation interaction had a detrimental effect on the binding with the targets. Given that these compounds consist of the indolo[1,2-*b*]isoquinoline scaffold, they were able to establish hydrophobic interactions with α-glucosidase. Therefore, van der Waals and nonpolar solvation energies emerged as the two crucial components of the overall binding free energy.

### 2.9. In Vitro Cytotoxicity

**Figure 11:**
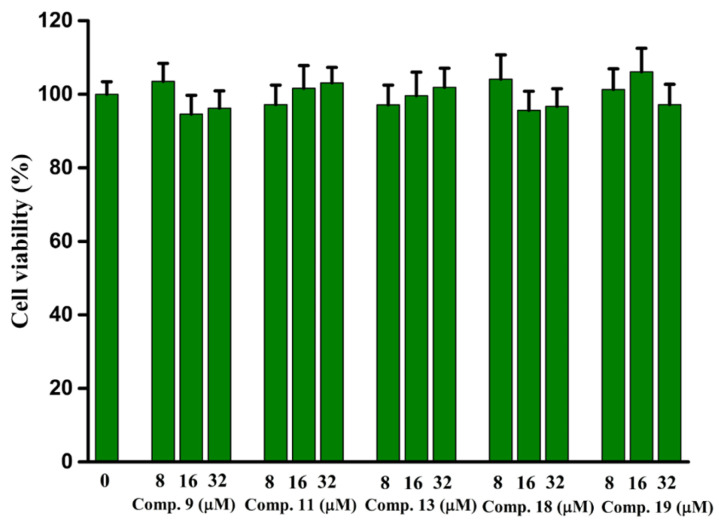
In vitro cytotoxicity of compounds **9**, **11**, **13**, **18**, and **19**.

The in vitro cytotoxicity of compounds **9**, **11**, **13**, **18**, and **19** against hepatocytes LO2 cells was detected using the MTT method. From Figure 11, compounds **9**, **11**, **13**, **18**, and **19** had no significant effect on cell viability up to a concentration of 32 μM, suggesting the safety of these compounds at low concentrations.

### 2.10. Drug-Like Properties

Finally, the drug-like properties of compounds **9**, **11**, **13**, **18**, and **19** were investigated using SwissADME software (https://www.swissadme.ch/index.php, accessed on 10 March 2023) and the results are summarized in Table 5. It can be seen that compounds **9**, **11**, **13**, **18**, and **19** show favorable drug properties, except for their poorly water solubility. The MW, RB, HBA, HBD, and TPSA values were within the scope (MW < 500, RB < 10, HBA < 5, HBD < 10, TPSA < 90 Å^2^), suggesting good drug-like properties of compounds **9**, **11**, **13**, **18**, and **19**. Molsoft software (https://molsoft.com/mprop/, accessed on 20 May 2023) and pkCSM software (https://biosig.lab.uq.edu.au/, accessed on 20 May 2023) were used to obtain other drug-like properties of compounds (Table 6 and Table 7), which also suggested favorable drug properties for compounds **9**, **11**, **13**, **18**, and **19**, including VDss, LogP, MolVol, BBB, and MolLPSA properties.

## 3. Experimental

### 3.1. Synthesis of Indolo[1,2-b]isoquinoline Derivatives (**1**–**20**)

**a** (120.6 mg, 0.3 mmol, 1 equiv), **b** (36.9 mg, 0.36 mmol, 1.2 equiv), Pd_2_(dba)_3_ (6.9 mg, 0.0075 mmol, 2.5 mol%), P(4-OMePh)_3_ (10.6 mg, 0.03 mmol, 10 mol%), LiCl (12.7 mg, 0.3 mmol, 1.0 equiv), NEt_3_ (0.25 mL, 1.8 mmol, 6.0 equiv) were transferred into a 15 mL tube under N_2_ atmosphere. Toluene (2 mL) was added to the reaction tube. To an *In-Ex* tube, Et_3_N (276 μL, 2 mmol) and HCO_2_H/Ac_2_O (266 μL, 2 mmol) were added, and the vial was sealed and placed in a heating block that was preheated to 120 °C. After a period of 12 h, the reaction vial was cooled to room temperature. Then, the solution was extracted with EtOAc. The combined organic phase was dried over Na_2_SO_4_ and concentrated under reduced pressure. The residue was purified by flash chromatography on silica gel diluted with petroleum ether/EtOAc (*v*/*v* = 50:1 to 10:1) to afford the products indolo[1,2-b]isoquinoline derivatives (**1**~**20**) [25].

**(Compound 1). ^1^H NMR (500 MHz, CDCl_3_)** δ 8.74 (d, *J* = 8.2 Hz, 1H), 8.57–8.50 (m, 1H), 7.70–7.62 (m, 2H), 7.62–7.54 (m, 2H), 7.53–7.46 (m, 3H), 7.3–7.32 (m, 1H), 7.32–7.28 (m, 2H), 7.25 (dd, *J* = 12.2, 4.7 Hz, 1H). **^13^C NMR (126 MHz, CDCl_3_)** δ 182.7, 159.0, 146.9, 136.6, 136.5, 133.1, 131.8, 130.4, 130.3, 129.8, 129.6, 128.8, 128.7, 128.6, 128.6, 126.5, 126.1, 124.9, 124.3, 118.3.

**(Compound 2). ^1^H NMR (500 MHz, CDCl_3_)** δ 8.78 (d, *J* = 8.2 Hz, 1H), 8.59–8.53 (m, 1H), 7.69 (m, 6.6 Hz, 2H), 7.66–7.58 (m, 2H), 7.46–7.42 (m, 1H), 7.37 (d, *J* = 7.9 Hz, 2H), 7.28 (dd, *J* = 15.5, 8.0 Hz, 3H), 2.49 (s, 3H). **^13^C NMR (126 MHz, CDCl_3_)** δ 182.8, 159.0, 146.9, 138.8, 136.7, 136.6, 133.1, 130.3, 129.8, 129.6, 129.5, 129.0, 128.7, 128.6, 128.6, 126.9, 126.1, 125.0, 124.4, 118.4, 21.6.

**(Compound 3). ^1^H NMR (500 MHz, CDCl_3_)** δ 8.80 (d, *J* = 8.2 Hz, 1H), 8.63–8.55 (m, 1H), 7.75–7.67 (m, 2H), 7.67–7.59 (m, 2H), 7.43 (m, 2H), 7.35 (d, *J* = 7.8 Hz, 1H), 7.30 (t, *J* = 7.4 Hz, 1H), 7.17 (d, *J* = 7.2 Hz, 2H), 2.45 (s, 3H). **^13^C NMR (126 MHz, CDCl_3_)** δ 182.8, 159.1, 146.9, 138.4, 136.6, 133.2, 131.8, 130.3, 129.7, 129.7, 129.0, 128.7, 128.6, 126.9, 126.2, 125.0, 124.4, 118.4, 21.7.

**(Compound 4). ^1^H NMR (500 MHz, CDCl_3_)** δ 8.80 (d, *J* = 8.2 Hz, 1H), 8.59 (dd, *J* = 7.8, 1.2 Hz, 1H), 7.75–7.68 (m, 2H), 7.64 (m, 2H), 7.48–7.33 (m, 3H), 7.31 (m, 1H), 7.28–7.24 (m, 1H), 7.19 (dd, *J* = 7.5, 0.9 Hz, 1H), 2.09 (s, 3H). **^13^C NMR (126 MHz, CDCl_3_)** δ 182.7, 159.1, 147.1, 136.9, 136.7, 136.2, 133.4, 131.6, 130.5, 129.7, 129.6, 129.0, 128.8, 128.7, 128.5, 126.3, 126.2, 125.9, 124.8, 124.4, 118.3, 19.8.

**(Compound 5). ^1^H NMR (500 MHz, CDCl_3_)** δ 8.78 (d, *J* = 8.2 Hz, 1H), 8.58–8.54 (m, 1H), 7.69 (m, 2H), 7.65–7.58 (m, 2H), 7.45 (dd, *J* = 6.6, 2.5 Hz, 1H), 7.39 (d, *J* = 7.9 Hz, 2H), 7.32–7.26 (m, 3H), 2.80 (q, *J* = 7.6 Hz, 2H), 1.36 (t, *J* = 7.6 Hz, 3H). **^13^C NMR (126 MHz, CDCl_3_)** δ 182.8, 159.0, 146.9, 144.9, 136.7, 136.6, 133.1, 130.3, 129.8, 129.6, 129.0, 128.9, 128.6, 128.6, 128.2, 126.9, 126.1, 125.0, 124.3, 118.4, 28.9, 15.4.

**(Compound 6). ^1^H NMR (500 MHz, CDCl_3_)** δ 8.79 (d, *J* = 8.2 Hz, 1H), 8.59–8.55 (m, 1H), 7.75–7.66 (m, 2H), 7.66–7.59 (m, 2H), 7.57 (d, *J* = 8.3 Hz, 2H), 7.49–7.44 (m, 1H), 7.34–7.27 (m, 3H), 1.43 (s, 9H). **^13^C NMR (126 MHz, CDCl_3_)** δ 182.9, 159.1, 151.8, 146.9, 136.7, 136.6, 133.1, 130.3, 130.3, 129.7, 129.6, 129.1, 128.7, 128.6, 127.0, 126.2, 125.7, 125.0, 124.3, 118.4, 34.9, 31.5.

**(Compound 7). ^1^H NMR (500 MHz, CDCl_3_)** δ 8.78 (d, *J* = 8.2 Hz, 1H), 8.59–8.56 (m, 1H), 7.74–7.68 (m, 2H), 7.68–7.62 (m, 2H), 7.54 (d, *J* = 8.3 Hz, 2H), 7.38 (dd, *J* = 6.9, 2.0 Hz, 1H), 7.35–7.29 (m, 3H). **^13^C NMR (126 MHz, CDCl_3_)** δ 182.8, 159.0, 147.0, 136.9, 136.2, 135.1, 133.3, 131.4, 130.5, 130.3, 129.7, 129.2, 128.8, 128.8, 128.6, 126.3, 125.2, 124.8, 124.5, 118.4.

**(Compound 8). ^1^H NMR (500 MHz, CDCl_3_)** δ 8.81 (d, *J* = 8.2 Hz, 1H), 8.61–8.58 (m, 1H), 7.77–7.68 (m, 2H), 7.68–7.61 (m, 2H), 7.51–7.47 (m, 1H), 7.34–7.28 (m, 3H), 7.12–7.06 (m, 2H), 3.92 (s, 3H). **^13^C NMR (126 MHz, CDCl_3_)** δ 182.9, 160.1, 159.1, 146.9, 136.8, 136.7, 133.2, 131.3, 130.4, 129.7, 129.0, 128.7, 128.7, 126.7, 126.2, 125.1, 124.4, 123.6, 118.4, 114.3, 55.5.

**(Compound 9). ^1^H NMR (500 MHz, CDCl_3_)** δ 8.73 (d, *J* = 8.2 Hz, 1H), 8.56–8.47 (m, 1H), 7.67 (dd, *J* = 7.5, 0.6 Hz, 1H), 7.65–7.60 (m, 1H), 7.60–7.55 (m, 2H), 7.54 (dd, *J* = 8.8, 4.2 Hz, 1H), 7.26–7.17 (m, 4H), 6.85 (d, *J* = 5.3 Hz, 2H), 3.01 (s, 6H). **^13^C NMR (126 MHz, CDCl_3_)** δ 182.7, 159.1, 146.6, 136.4, 133.0, 131.1, 130.2, 129.9, 129.9, 129.6, 129.1, 128.5, 128.4, 126.0, 125.1, 124.2, 118.3, 29.7, 29.3.

**(Compound 10). ^1^H NMR (500 MHz, CDCl_3_)** δ 8.81 (d, *J* = 8.2 Hz, 1H), 8.62–8.59 (m, 1H), 7.77–7.70 (m, 2H), 7.69–7.59 (m, 3H), 7.51 (m, 1H), 7.46 (m, 1H), 7.36–7.30 (m, 2H), 7.28–7.25 (m, 1H). **^13^C NMR (126 MHz, CDCl_3_)** δ 182.7, 159.1, 147.3, 136.9, 135.6, 134.2, 133.4, 131.5, 131.3, 130.6, 130.5, 130.1, 129.7, 129.4, 128.9, 128.2, 127.4, 126.3, 124.7, 124.6, 123.1, 118.4.

**(Compound 11). ^1^H NMR (500 MHz, CDCl_3_)** ^1^H NMR (500 MHz, CDCl_3_) δ 8.77 (d, *J* = 8.5 Hz, 1H), 8.57 (dd, *J* = 7.9, 1.1 Hz, 1H), 7.97 (d, *J* = 8.4 Hz, 1H), 7.90 (d, *J* = 8.0 Hz, 1H), 7.82 (d, *J* = 9.9 Hz, 2H), 7.70–7.64 (m, 2H), 7.62 (m, 1H), 7.58–7.53 (m, 1H), 7.53–7.46 (m, 2H), 7.39 (ddd, *J* = 9.5, 6.0, 1.2 Hz, 2H), 7.25 (m, 1H). **^13^C NMR (126 MHz, CDCl_3_)** δ 182.8, 159.2, 147.1, 136.8, 136.7, 133.5, 133.4, 133.3, 130.5, 129.8, 129.4, 129.1, 128.9, 128.8, 128.5, 128.4, 128.1, 127.5, 127.5, 126.8, 126.6, 126.3, 125.0, 124.5, 118.5.

**(Compound 12). ^1^H NMR (500 MHz, CDCl_3_)** δ 8.78 (d, *J* = 8.1 Hz, 1H), 8.59 (d, *J* = 7.6 Hz, 1H), 8.24 (d, *J* = 8.0 Hz, 2H), 7.71 (t, *J* = 8.3 Hz, 2H), 7.69–7.60 (m, 2H), 7.48 (d, *J* = 8.0 Hz, 2H), 7.31 (t, *J* = 7.1 Hz, 2H), 3.99 (s, 3H). **^13^C NMR (126 MHz, CDCl_3_)** δ 182.7, 166.9, 158.9, 147.1, 136.9, 136.9, 135.9, 133.4, 130.6, 130.6, 130.2, 130.1, 129.6, 128.8, 128.7, 128.5, 126.3, 125.3, 124.7, 124.5, 118.4, 52.4.

**(Compound 13). ^1^H NMR (500 MHz, CDCl_3_)** δ 8.76 (d, *J* = 8.3 Hz, 1H), 8.57 (dd, *J* = 7.6, 1.5 Hz, 1H), 7.71–7.62 (m, 4H), 7.61–7.54 (m, 3H), 7.44–7.40 (m, 1H), 7.40–7.34 (m, 2H). **^13^C NMR (126 MHz, CDCl_3_)** δ 181.5, 159.0, 145.2, 136.5, 136.3, 133.4, 132.1, 131.6, 130.7, 129.8, 129.6, 129.2, 129.1, 128.9, 128.8, 127.5, 126.3, 124.2, 119.6.

**(Compound 14). ^1^H NMR (500 MHz, CDCl_3_)** δ 8.57 (s, 1H), 8.53–8.49 (m, 1H), 7.62–7.51 (m, 3H), 7.51–7.46 (m, 3H), 7.32 (m, 3H), 7.04 (d, *J* = 7.8 Hz, 1H), 2.46 (s, 3H). **^13^C NMR (126 MHz, CDCl_3_)** δ 182.4, 159.1, 148.7, 147.4, 136.6, 133.2, 132.0, 130.3, 129.9, 129.6, 129.1, 128.9, 128.8, 128.8, 128.6, 127.3, 126.3, 124.3, 122.8, 118.8, 22.9.

**(Compound 15). ^1^H NMR (500 MHz, CDCl_3_)** δ 8.81 (d, *J* = 1.4 Hz, 1H), 8.58–8.48 (m, 1H), 7.66–7.55 (m, 3H), 7.54–7.47 (m, 3H), 7.38–7.33 (m, 1H), 7.30 (dd, *J* = 6.5, 2.8 Hz, 2H), 7.22 (dd, *J* = 8.1, 1.6 Hz, 1H). **^13^C NMR (126 MHz, CDCl_3_)** δ 181.5, 159.1, 147.4, 143.0, 136.5, 133.5, 131.6, 130.7, 129.8, 129.5, 129.1, 128.9, 128.8, 128.7, 127.2, 126.7, 125.3, 123.4, 118.9.

**(Compound 16). ^1^H NMR (500 MHz, CDCl_3_)** δ 8.82–8.68 (m, 1H), 8.52 (dd, *J* = 4.6, 1.7 Hz, 1H), 7.60 (dd, *J* = 13.9, 4.7 Hz, 2H), 7.50 (s, 2H), 7.32 (d, *J* = 18.4 Hz, 5H), 7.19 (s, 1H). **^13^C NMR (126 MHz, CDCl_3_)** δ 181.8 (d, *J _(C-F)_* = 2.5 Hz), 160.8 (d, *J _(C-F)_* = 248.2 Hz), 158.9, 143.3 (d, *J _(C-F)_* = 1.3 Hz), 136.4, 133.3, 131.6, 130.7, 129.8, 129.7, 129.1 (d, *J _(C-F)_* = 3.8 Hz), 128.9, 128.7, 127.5, 126.5 (d, *J _(C-F)_* = 7.6 Hz), 123.6, 123.4, 120.0 (d, *J _(C-F)_* = 7.6 Hz), 110.8, 110.6

**(Compound 17). ^1^H NMR (500 MHz, CDCl_3_)** δ 8.76 (d, *J* = 8.2 Hz, 1H), 8.44 (d, *J* = 8.1 Hz, 1H), 7.68 (m, 2H), 7.60–7.54 (m, 3H), 7.43 (dd, *J* = 8.1, 1.0 Hz, 1H), 7.41–7.35 (m, 2H), 7.30–7.25 (m, 1H), 7.15 (s, 1H), 2.37 (s, 3H). **^13^C NMR (126 MHz, CDCl_3_)** δ 182.8, 159.1, 147.0, 144.1, 136.6, 136.5, 132.0, 131.8, 129.9, 128.8, 128.8, 128.8, 128.6, 127.3, 127.6, 126.0, 124.9, 124.3, 118.3, 22.0.

**(Compound 18). ^1^H NMR (500 MHz, CDCl_3_)** δ 8.64 (d, *J* = 8.1 Hz, 1H), 8.08 (dd, *J* = 8.9, 2.6 Hz, 1H), 7.59 (dd, *J* = 13.9, 7.5 Hz, 2H), 7.48 (dd, *J* = 5.0, 1.4 Hz, 3H), 7.31 (ddd, *J* = 9.3, 7.2, 3.5 Hz, 3H), 7.21 (ddd, *J* = 17.6, 11.1, 4.6 Hz, 2H). **^13^C NMR (126 MHz, CDCl_3_)** δ 182.4, 163.7 (d, *J _(C-F)_* = 254.5 Hz), 157.9 (d, *J _(C-F)_* = 2.5 Hz), 146.6, 136.6, 133.0 (d, *J _(C-F)_* = 2.5 Hz), 131.8 (d, *J _(C-F)_* = 7.6 Hz), 131.6, 131.5 (d, *J _(C-F)_* = 7.6 Hz), 129.8, 129.1, 128.9, 128.1 (d, *J _(C-F)_* = 2.5 Hz), 126.4, 126.0, 124.9, 124.4, 121.4 (d, *J _(C-F)_* = 22.7 Hz), 118.3, 114.5 (d, *J _(C-F)_* = 23.9 Hz).

**(Compound 19). ^1^H NMR (500 MHz, CDCl_3_)** δ 8.55 (s, 1H), 8.38 (d, *J* = 8.1 Hz, 1H), 7.49 (ddd, *J* = 9.4, 6.0, 3.3 Hz, 4H), 7.37 (d, *J* = 8.1 Hz, 1H), 7.29 (dd, *J* = 7.1, 2.2 Hz, 2H), 7.07 (s, 1H), 7.01 (d, *J* = 7.7 Hz, 1H), 2.44 (s, 3H), 2.30 (s, 3H). **^13^C NMR (126 MHz, CDCl_3_)** δ 182.4, 159.2, 148.6, 147.4, 144.1, 136.6, 132.1, 131.7, 129.9, 129.3, 128.8, 128.7, 128.7, 128.6, 127.3, 127.1, 126.2, 124.2, 122.8, 118.8, 22.9, 22.0.

**(Compound 20). ^1^H NMR (500 MHz, CDCl_3_)** δ 8.71 (dd, *J* = 8.8, 4.1 Hz, 1H), 8.38 (d, *J* = 8.1 Hz, 1H), 7.54–7.48 (m, 3H), 7.40 (d, *J* = 8.3 Hz, 1H), 7.30 (dd, *J* = 14.3, 4.4 Hz, 5H), 7.08 (s, 1H), 2.32 (s, 3H). **^13^C NMR (126 MHz, CDCl_3_)** δ 181.8(d, *J _(C-F)_* = 2.5 Hz), 160.7 (d, *J _(C-F)_* = 248.2 Hz), 158.9, 144.3, 143.2, 136.4, 132.1, 131.8, 129.8, 129.0 (d, *J _(C-F)_* = 1.3 Hz), 128.9, 128.7, 127.5, 127.4, 126.5 (d, *J _(C-F)_* = 7.6 Hz), 123.5, 123.3, 119.9 (d, *J _(C-F)_* = 7.6 Hz), 110.7, 110.5, 22.0.

### 3.2. Materials and Methods

*α*-Glucosidase from *Saccharomyces cerevisiae* (EC 3.2.1.20) was purchased from Sigma-Aldrich. *p*-Nitrophenyl-*α*-D-galactopyranoside (PNPG) was obtained from Abcam. Other reagents were commercially available.

### 3.3. α-Glucosidase Inhibition and Kinetics Assay

The α-glucosidase inhibitory activity of indole[1,2-b]isoquinoline derivatives (**1**–**20**) was determined using PNPG as a substrate according to previous reports [34,35,36]. α-Glucosidase (0.1 U/mL) and the test compound were added to phosphate-buffered saline (0.1 M, pH 6.8) and then incubated for 10 min at 37 °C. After the addition of PNPG (0.25 mM), the change in absorbance was measured at 405 nm. The test compounds were dissolved in DMSO and acarbose was used as a positive control. All experiments were performed four times.

The kinetics of enzyme inhibition for compounds **9**, **11**, **13**, **18**, and **19** were obtained by plotting the enzymatic reaction rate versus enzyme concentration with or without compounds **9**, **11**, **13**, **18**, and **19**, and the kinetics of substrate inhibition were measured using Lineweaver Burk plots of the enzymatic reaction rate versus substrate concentration with or without compounds **9**, **11**, **13**, **18**, and **19** [37,38,39,40].

### 3.4. D Fluorescence Spectra

Compounds **9**, **11**, **13**, **18**, and **19** were added to α-glucosidase (5 μM) in 100 μL of PBS and incubated for 5 min, then the 3D fluorescence spectra of the mixture were recorded [32]^.^ The excitation and emission wavelengths were 200–500 nm and the slit width was 2.5 nm. All data were imported into Matlab for processing.

### 3.5. CD Spectra

Compounds **9**, **11**, **13**, **18**, and **19** with different concentrations were added into α-glucosidase solution (31 μM) and incubated for 5 min, respectively, then the CD spectra of the mixture were recorded [33]. CDNN was used to analyze the ratio of protein secondary conformations.

### 3.6. Molecular Docking 

Molecular docking between compounds **9**, **11**, **13**, **18**, and **19** and α-glucosidase was conducted using SYBYL software [41,42,43]. The homology model of α-glucosidase had been built in our previous works. Compounds **9**, **11**, **13**, **18**, and **19** were built and treated with energy minimization. α-Glucosidase was also optimized using the internal program, followed by the generation of the active pocket. Molecular docking was conducted in the default format.

**Figure 12:**
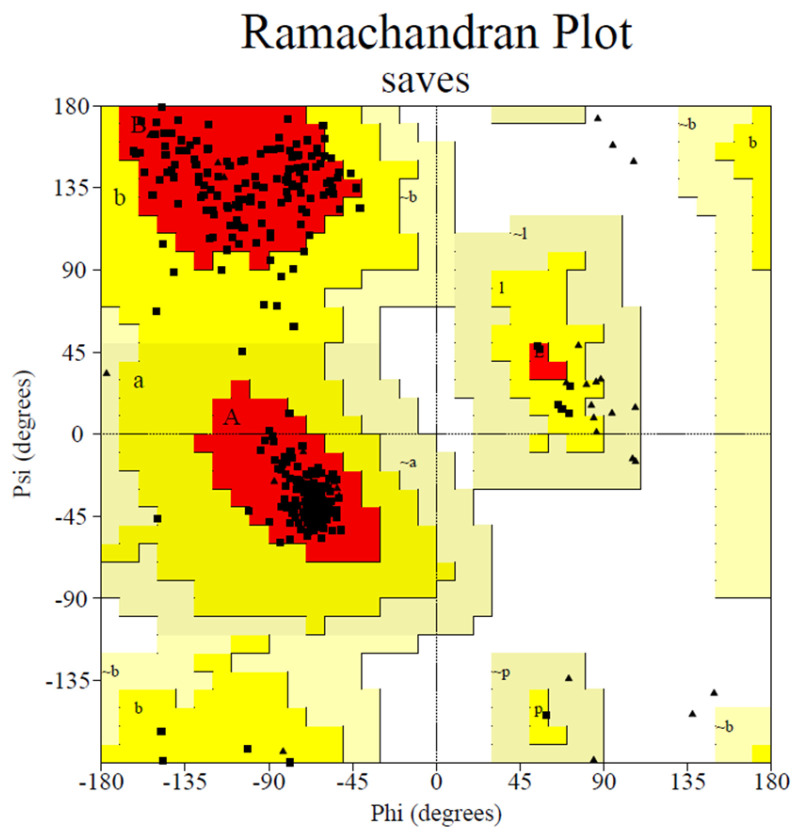
Ramachandran plot results of the homology model of human α-glucosidase.

For the homology model of human α-glucosidase, the sequence in FASTA format was obtained from UniProt (ID O33830), and alpha-glucosidase A (ID: 1OBB) was selected as the template. The human α-glucosidase homology models were constructed using Modeler 10.1 software (http://salilab.org/modeller/, accessed on 20 June 2023). Then, their qualities were verified by using a Ramachandran plot (http://services.mbi.ucla.edu/PROCHECK/, accessed on 20 June 2023). The optimal homology mode with a Phi (degrees) of 93.3% (Figure 12) was selected for subsequent docking.

### 3.7. Molecular Dynamics Simulation

A molecular dynamics simulation was carried out to analyze the protein backbone stability in docking compounds **9**, **11**, **13**, **18**, and **19** using the Desmond simulation. The complex was treated with internal procedures, including filling water, cleaning total charge, and minimizing energy. Then, the dynamics simulation was run with a simulation length of 100 ns and a relaxation time of 1 ps. 

### 3.8. MTT Assay 

The in vitro cytotoxicity of compound **11** against hepatocytes LO2 cells was assayed using the MTT method [44,45]. LO2 cells were cultured in DMEM containing 10% FBS, 100 IU/mL penicillin, and 100 IU/mL streptomycin at 37 °C under 5% CO2. An amount of 100 μL of LO2 cells was seeded into a 96-well plate (5000 per well) for 24 h, and was then treated with compounds **9**, **11**, **13**, **18**, and **19** for 24 h, respectively. Then, MTT solution was added into each well and incubated for 4 h. An amount of 100 mL of DMSO was used to dissolve the obtained crystallization. Then, this absorbance was determined at 490 nm. Each sample was performed in triplicate.

## Figures and Tables

**Table 1:** α-Glucosidase inhibitory activity of all synthesized compounds.

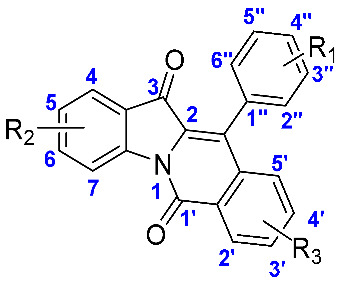				
**Compound**	**R_1_**	**R_2_**	**R_3_**	**IC_50_ (μM)**
**1**	H	H	H	23.46 ± 0.23
**2**	4-CH_3_	H	H	17.37 ± 0.25
**3**	3-CH_3_	H	H	9.25 ± 0.14
**4**	2-CH_3_	H	H	22.25 ± 0.63
**5**	4-CH_2_CH_3_	H	H	16.08 ± 0.46
**6**	4-C(CH_3_)_3_	H	H	5.76 ± 0.26
**7**	4-Cl	H	H	14.66 ± 0.37
**8**	4-OCH_3_	H	H	15.06 ± 0.44
**9**	4-N(CH_3_)_2_	H	H	40.92 ± 0.69
**10**	2-Cl	H	H	12.16 ± 0.46
**11**	naphthalene	H	H	3.44 ± 0.36
**12**	4-COOCH_3_	H	H	32.79 ± 0.72
**13**	H	5-Cl	H	6.35 ± 0.25
**14**	H	6-CH_3_	H	17.02 ± 0.37
**15**	H	6-Cl	H	9.25 ± 0.41
**16**	H	5-F	H	10.15 ± 0.36
**17**	H	H	4-CH_3_	7.95 ± 0.43
**18**	H	H	3-F	7.97 ± 0.31
**19**	H	6-CH_3_	4-CH_3_	41.24 ± 0.76
**20**	H	5-F	4-CH_3_	11.87 ± 0.36
**Acarbose**				640.57 ± 5.13

**Table 2:** The inhibition kinetics constants of compounds **9**, **11**, **13**, **18**, and **19**.

Compound	*K*_i_ (μM)	*K*_is_ (μM)	*K*_m_ (μM)	*V*_max_ (μM·min^−1^·mg^−1^)
**9**	30.46	96.19	1.67	0.12
**11**	0.46	14.57	0.12	0.06
**13**	3.62	11.65	0.60	0.08
**18**	4.62	12.34	0.49	0.08
**19**	8.38	42.30	0.01	0.05

**Table 3:** The secondary structure contents of α-glucosidase with compounds **9**, **11**, **13**, **18**, and **19** complexes.

Comp.	Molar Ratio [α-Glu]:[Comp.]	α-Helix (%)	β-Sheet (%)	β-Turn (%)	Rndm Coil (%)
**9**	1:0	9.40	38.40	19.50	35.40
	1:1	10.80	35.60	19.30	35.10
	1:2	13.00	31.90	19.30	34.60
	1:3	13.10	31.70	19.10	33.40
**11**	1:0	8.50	34.10	19.80	35.50
	1:1	8.40	34.30	20.00	35.60
	1:2	8.20	34.70	20.00	35.60
	1:3	7.80	35.20	20.10	35.80
**13**	1:0	9.40	38.40	19.50	35.40
	1:1	8.80	40.60	19.30	35.60
	1:2	9.10	39.60	19.40	35.40
	1:3	11.20	34.60	19.60	35.10
**18**	1:0	9.40	38.40	19.50	35.40
	1:1	11.10	35.30	19.40	35.00
	1:2	11.20	35.00	19.30	35.00
	1:3	12.80	35.00	19.30	34.70
**19**	1:0	9.40	38.40	19.50	35.40
	1:1	12.90	31.80	19.40	34.60
	1:2	11.50	34.20	19.30	34.70
	1:3	12.80	34.40	19.30	34.90

**Table 4:** Predicted docking energies, total binding free energy and its primary components of compounds **9**, **11**, **13**, **18**, and **19**.

Compd.	ΔG_bind_ (kcal/mol)	ΔE_VDW_ (kcal/mol)	ΔE_ele_ (kcal/mol)	ΔE_GB_ (kcal/mol)	ΔE_GA_ (kcal/mol)
**9**	−47.23 ± 5.09	−42.34 ± 2.31	−6.89 ± 1.71	26.57 ± 5.10	−14.76 ± 2.04
**11**	−66.94 ± 4.24	−46.50 ± 2.22	−8.60 ± 2.89	21.62 ± 2.72	−26.42 ± 1.71
**13**	−60.27 ± 3.20	−45.21 ± 1.78	−0.78 ± 1.55	18.45 ± 1.84	−24.08 ± 1.18
**18**	−52.87 ± 5.81	−42.34 ± 2.31	−12.64 ± 3.99	25.44 ± 1.47	−14.76 ± 2.03
**19**	−51.49 ± 4.20	−35.25 ± 2.46	−3.73 ± 3.40	12.92 ± 1.86	−20.70 ± 1.29

ΔG_bind_ (free energy of binding), ΔE_VDW_ (van der Waals energy), ΔE_ele_ (electrostatic energy), ΔE_GB_ (polar solvation energy), ΔE_GA_ (nonpolar solvation energy).

**Table 5:** Drug-like properties of compounds **9**, **11**, **13**, **18**, and **19** by SwissADME software.

Comp.	MW	RB	HBA	HBD	PPSA	Log Po/w	WS
**9**	373.4	1	2	0	39.07	4.83	Poorly
**11**	373.4	1	2	0	39.07	4.83	Poorly
**13**	357.79	1	2	0	39.07	4.46	Poorly
**18**	341.33	1	3	0	39.07	4.23	Poorly
**19**	351.40	1	2	0	39.07	4.61	Poorly

MW (molecular weight, g/mol), RB (rotatable bonds), HBA (h-bond acceptor atoms), HBD (h-bond donor atoms), TPSA (topology polar surface area, Å^2^), WS (water solubility).

**Table 6:** Drug-like properties of compound **11** using Molsoft software.

MF	MW	RB	HBA	HBD	MolVol	BBB	MolLPSA
**9**	373.4	1	2	0	262.08 A^3^	4.10	27.92 A^2^
**11**	373.11	1	2	0	262.08 A^2^	4.10	27.92 A^2^
**13**	357.79	1	2	0	357.74 A^3^	4.69	38.19 A^2^
**18**	341.33	1	2	0	342.46 A^3^	4.69	28.19 A^2^
**19**	1	2	0	378.50 A^3^	4.66	28.19 A^2^	1

MolVol (molar volume), BBB (Blood Brain Barrier), MolLPSA (polar surface area).

**Table 7:** Drug-like properties of compound **11** using pkCSM software.

MF	MW	RB	Acceptors	Donors	VDss	Log P	WS
**9**	373.4	1	3	0	−0.61	5.355	−8.256
**11**	373.4	1	3	0	−0.61	5.355	−8.256
**13**	357.79	1	3	0	−0.511	4.8555	−4.52
**18**	341.33	1	3	0	−0.604	4.3412	−4.522
**19**	351.40	1	3	0	−0.363	4.81894	−5.622

VDss (volume of distribution steady-state), Acceptors (hydrogen acceptor), Donors (hydrogen donor).

## Data Availability

Not applicable.

## Supplementary Materials

The following supporting information can be downloaded at: https://www.mdpi.com/article/10.3390/molecules28135282/s1. NMR data.

## Sample Availability

Compound samples are available from the authors.
