# Supplementary material for: Anti-α-Glucosidase, SAR Analysis, and Mechanism Investigation of Indolo[1,2-b]isoquinoline Derivatives
Source: Molecules. 2023 Jul 7;28(13):5282. doi: 10.3390/molecules28135282 (PMC10343752; doi:10.3390/molecules28135282)
Supplement: Supplementary file 1 [file molecules-28-05282-s001.zip › molecules-2382486-supplementary.pdf]

## Supplementary File (NMR data)

# Anti-α-Glucosidase, SAR Analysis, and Mechanism Investigation of Indolo[1,2-b]isoquinoline Derivatives

**Mengyue Li, Lin Li, Li Lu, Xuetao Xu *, Jinhui Hu and Jin-Bao Peng ***

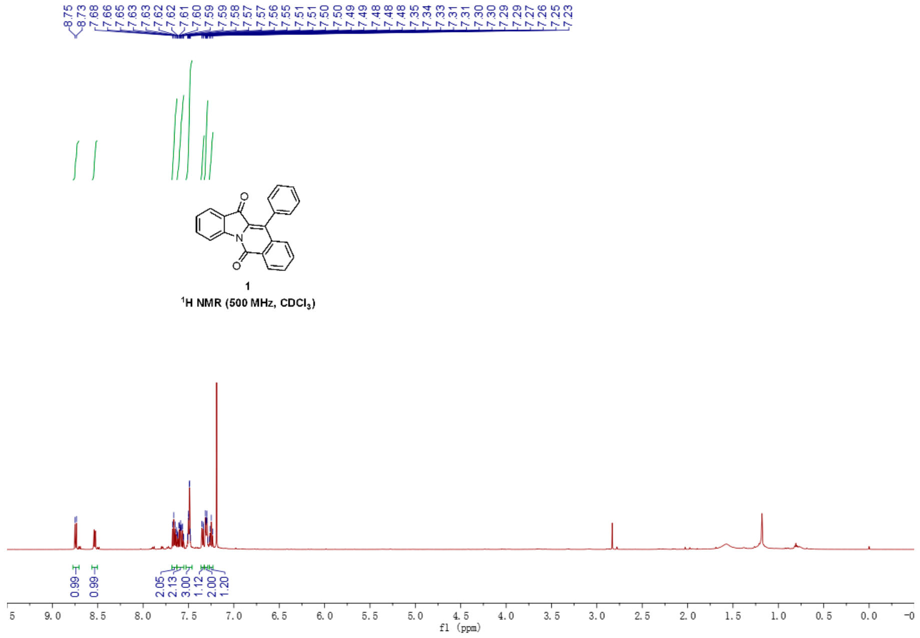


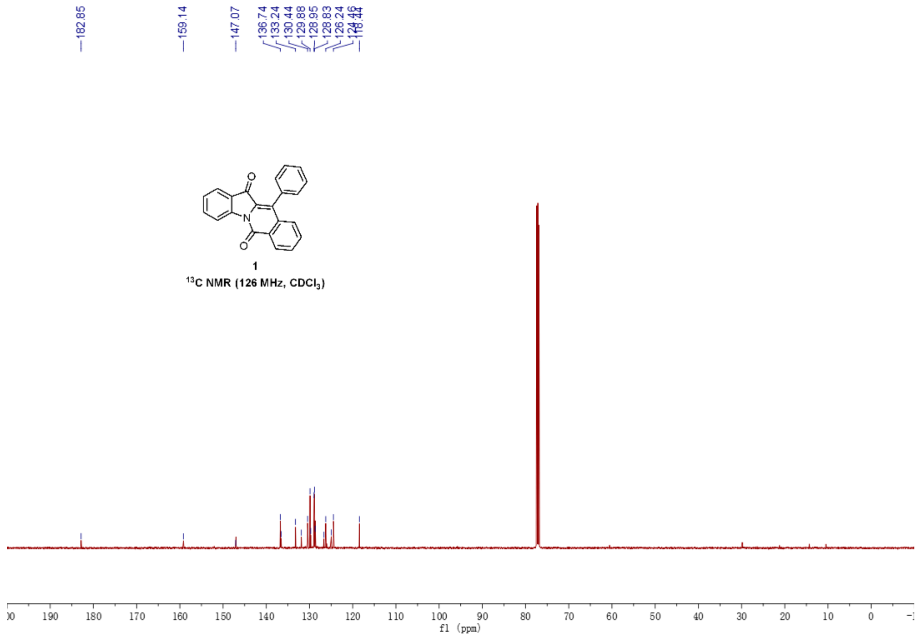

$^1$H NMR and $^{13}$C NMR data of compound **1**.

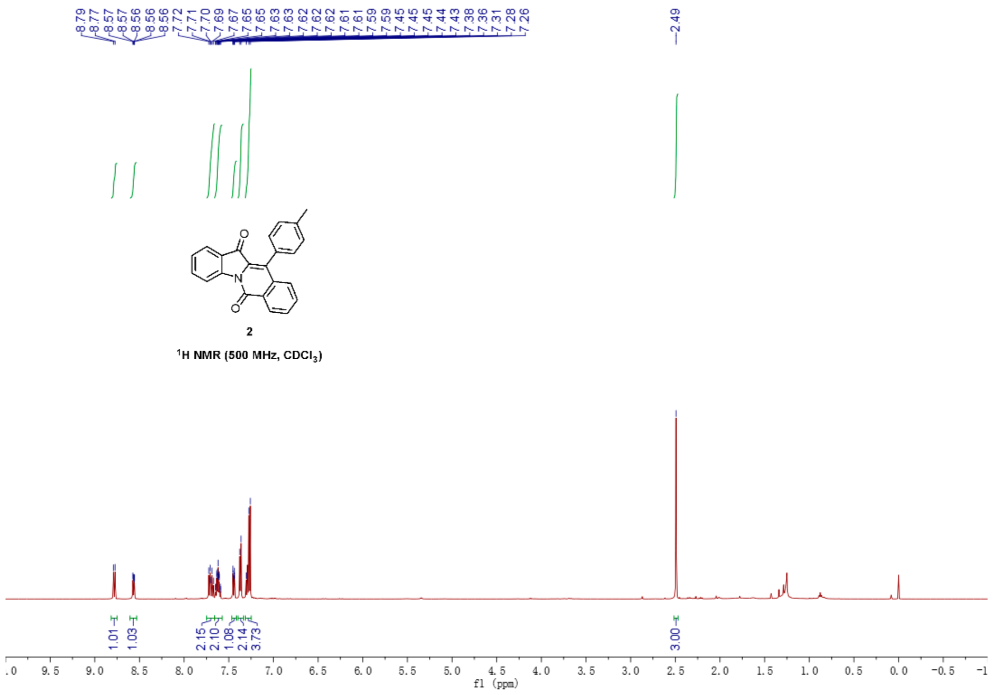


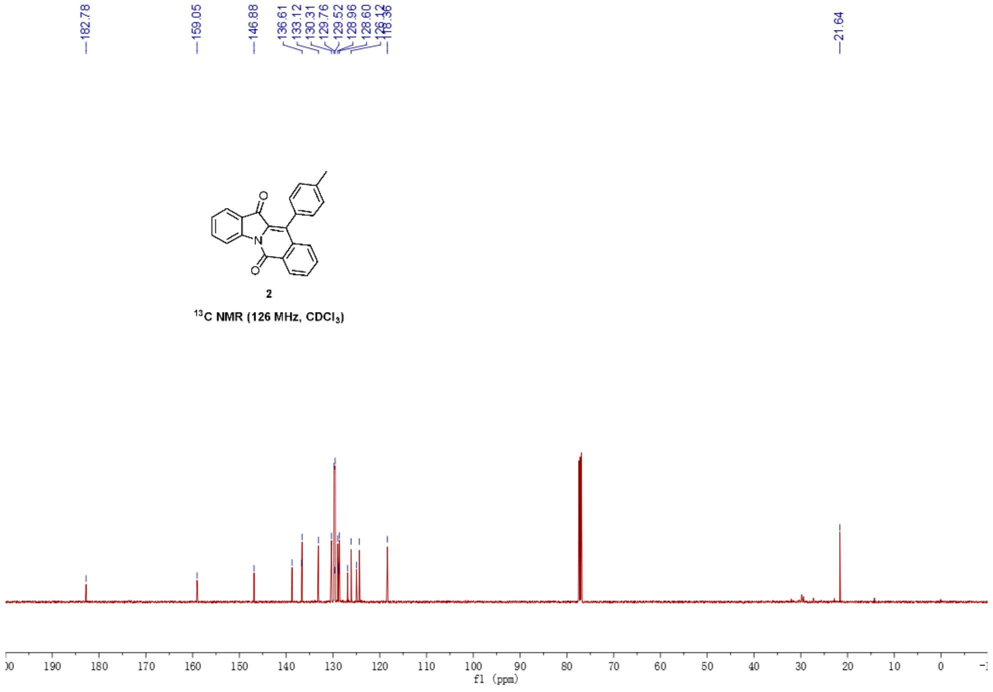


$^1$H NMR and $^{13}$C NMR data of compound **2**.

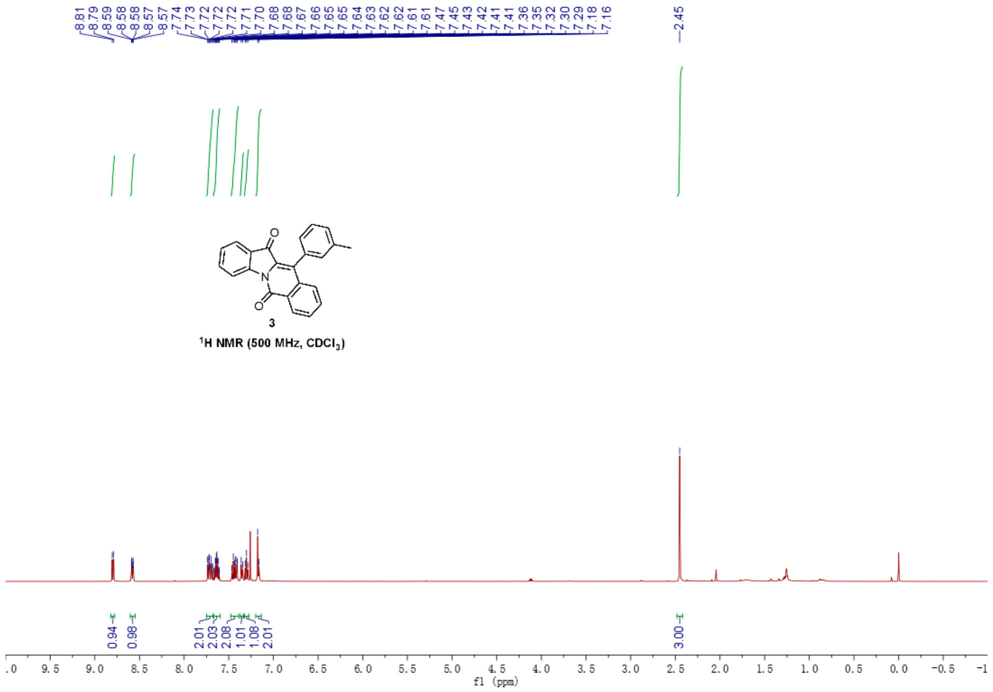


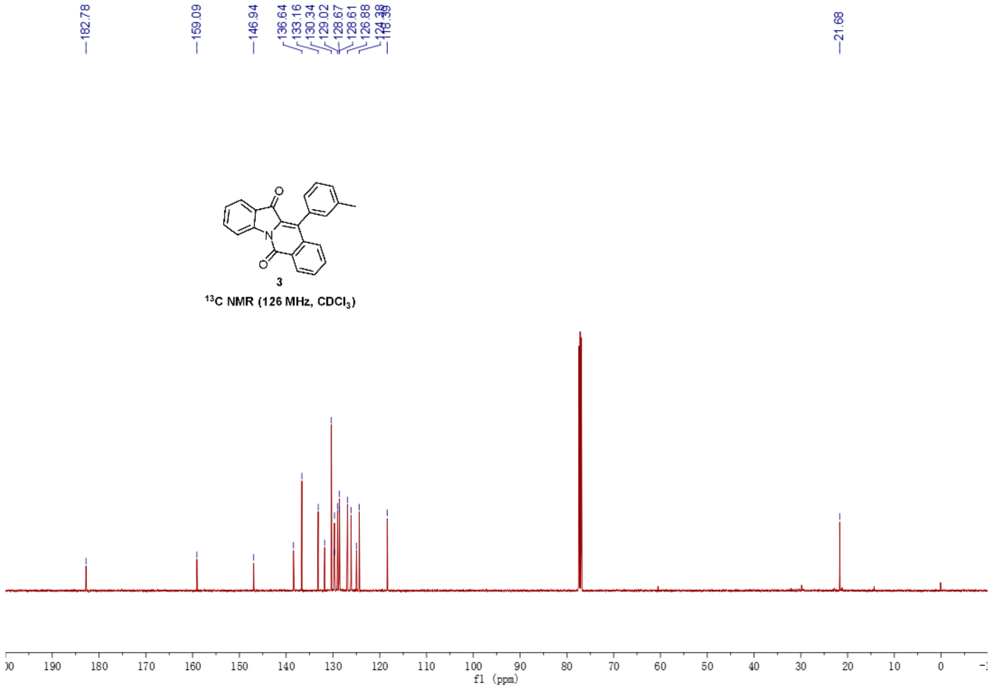


$^{1}$H NMR and $^{13}$C NMR data of compound **3**.

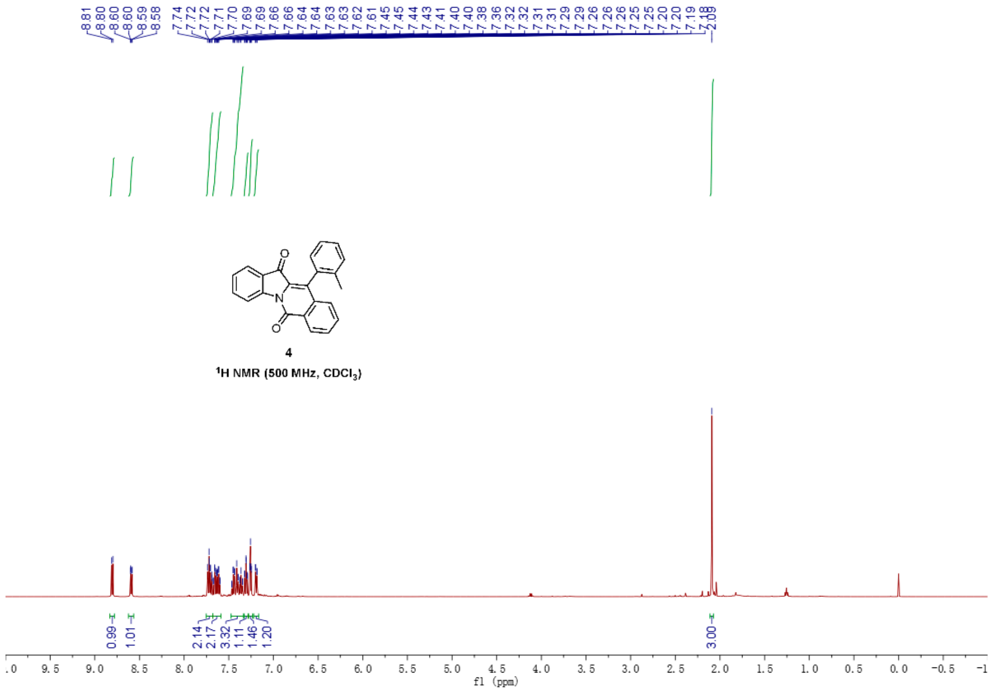


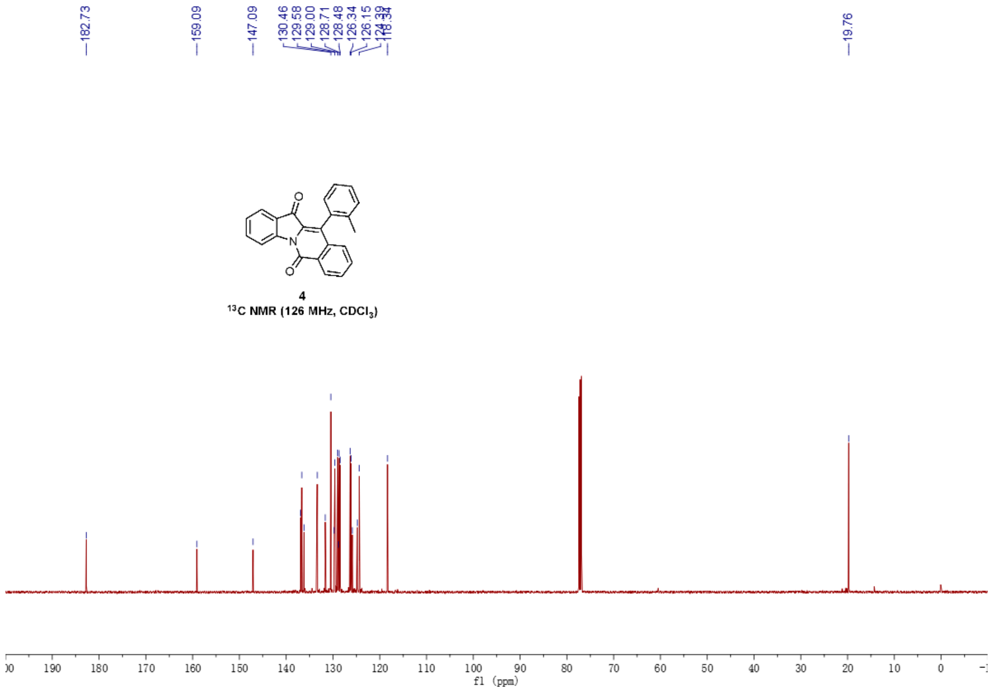


$^{1}$H NMR and $^{13}$C NMR data of compound **4**.

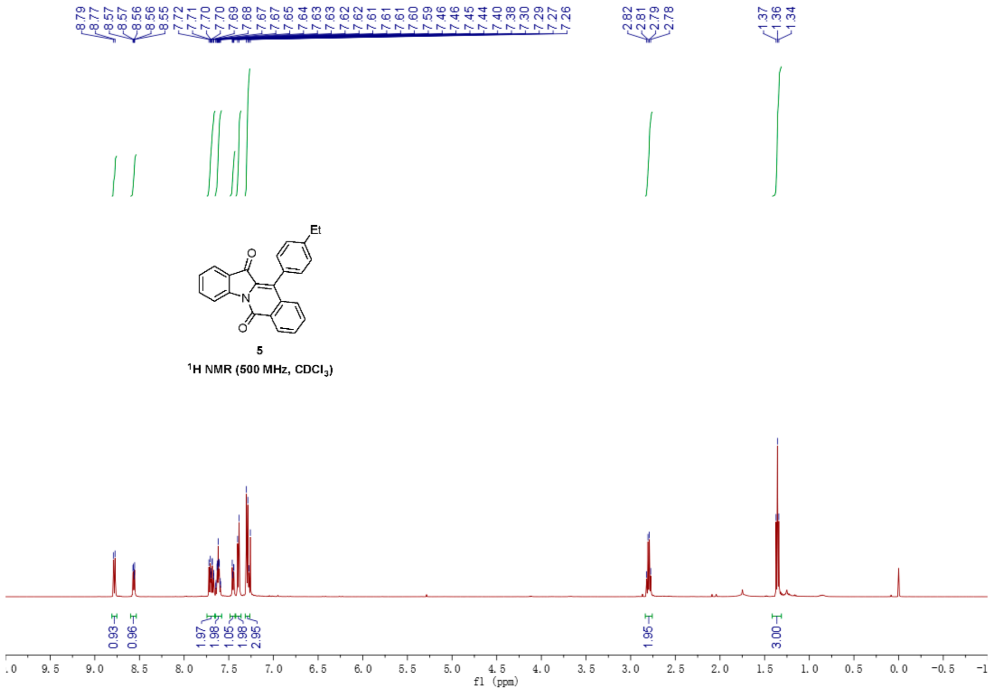


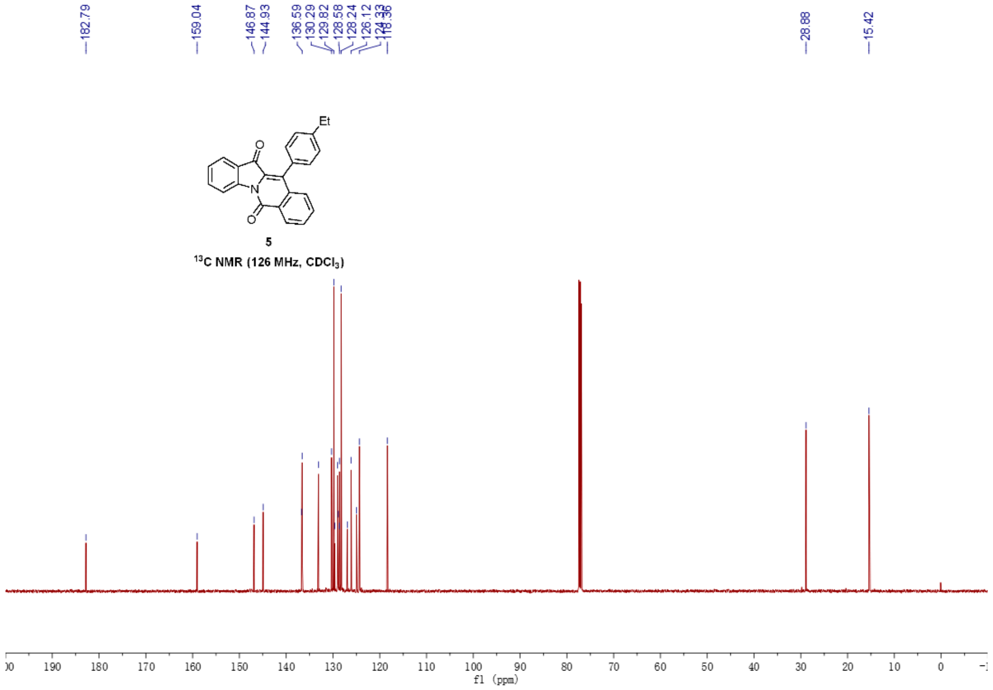


$^{1}$H NMR and $^{13}$C NMR data of compound **5**.

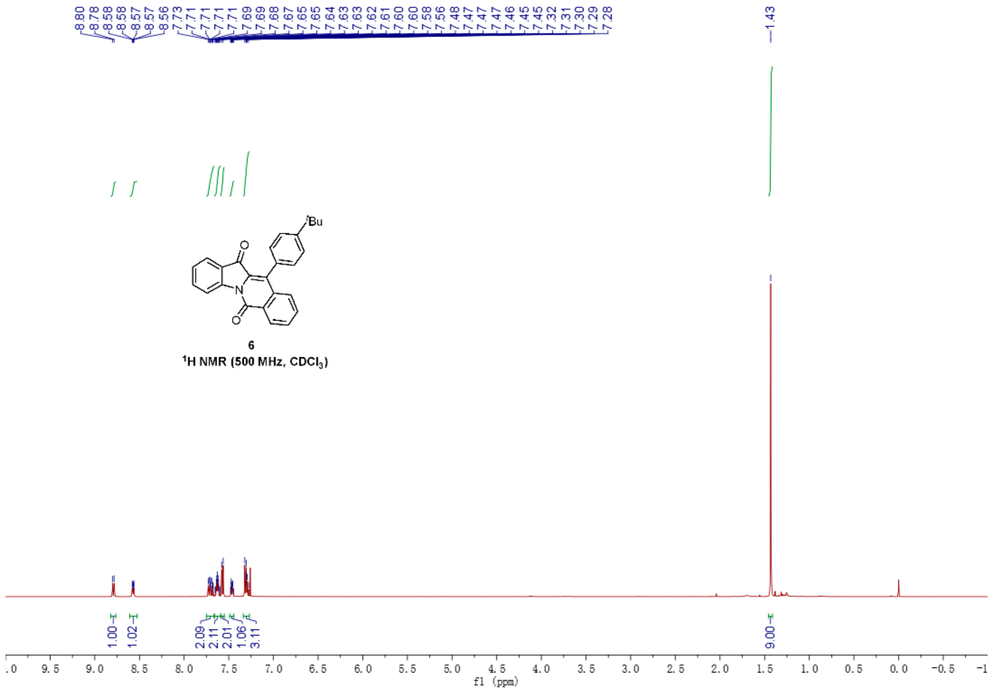


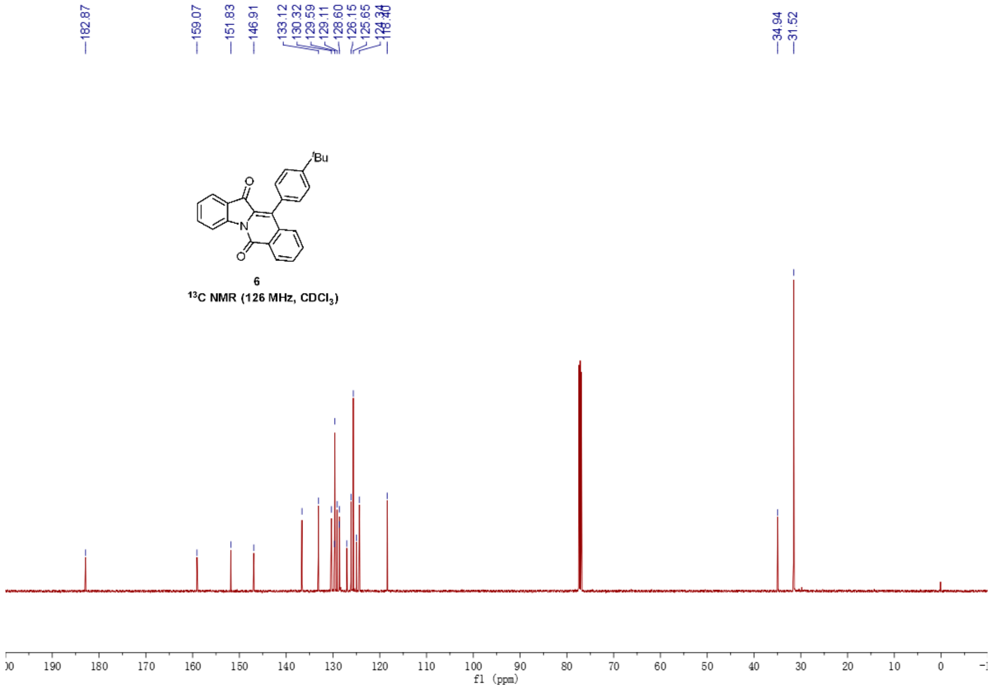


$^1$H NMR and $^{13}$C NMR data of compound **6**.

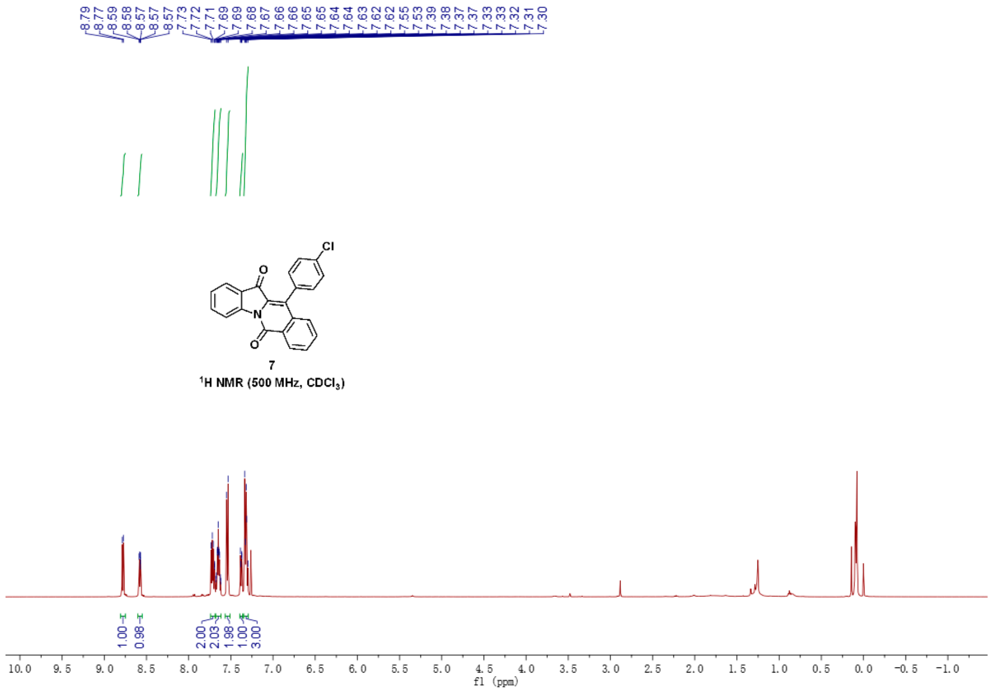


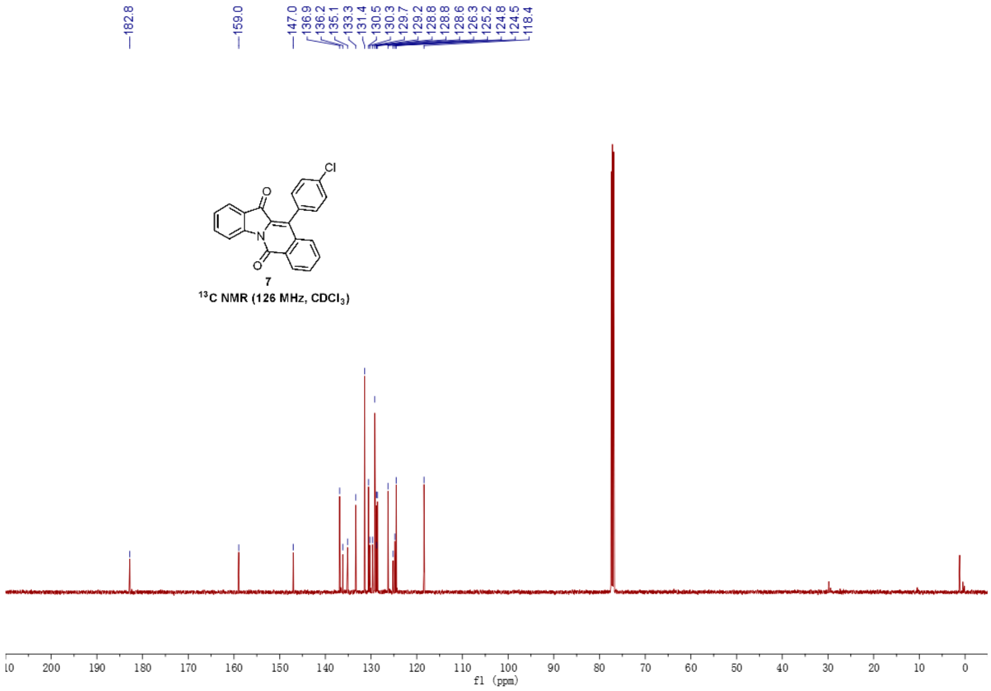


$^{1}$H NMR and $^{13}$C NMR data of compound **7**.

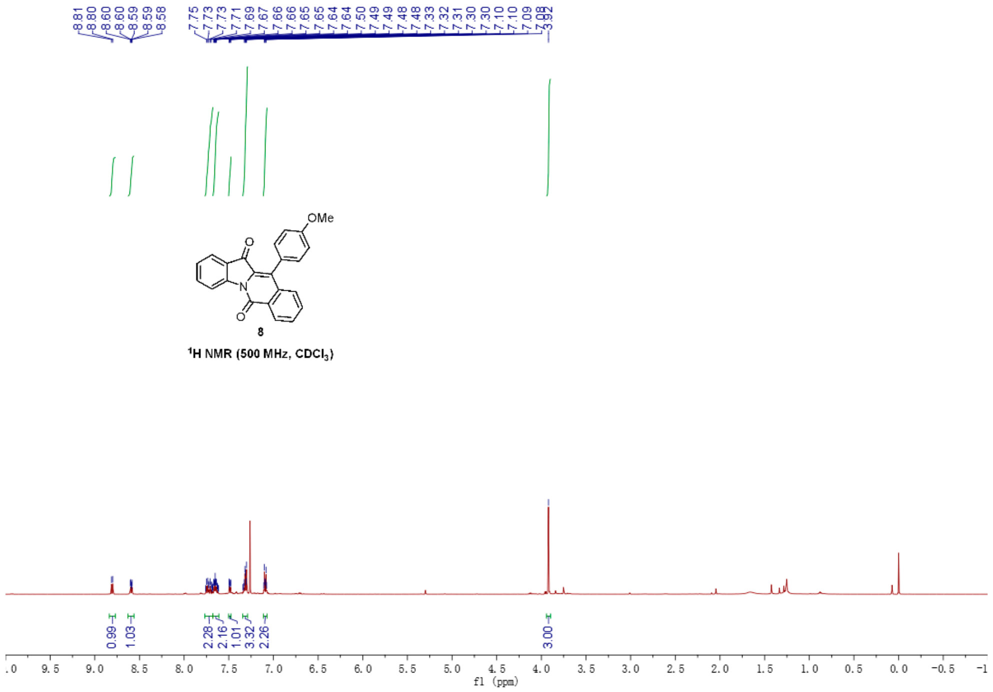

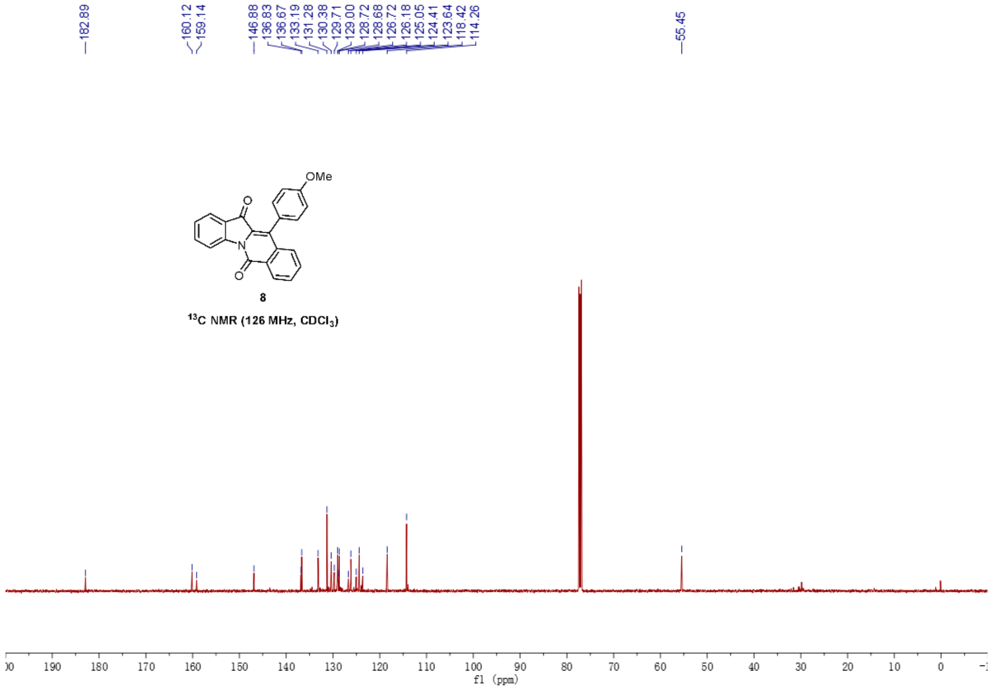

$^{1}$H NMR and $^{13}$C NMR data of compound **8**.

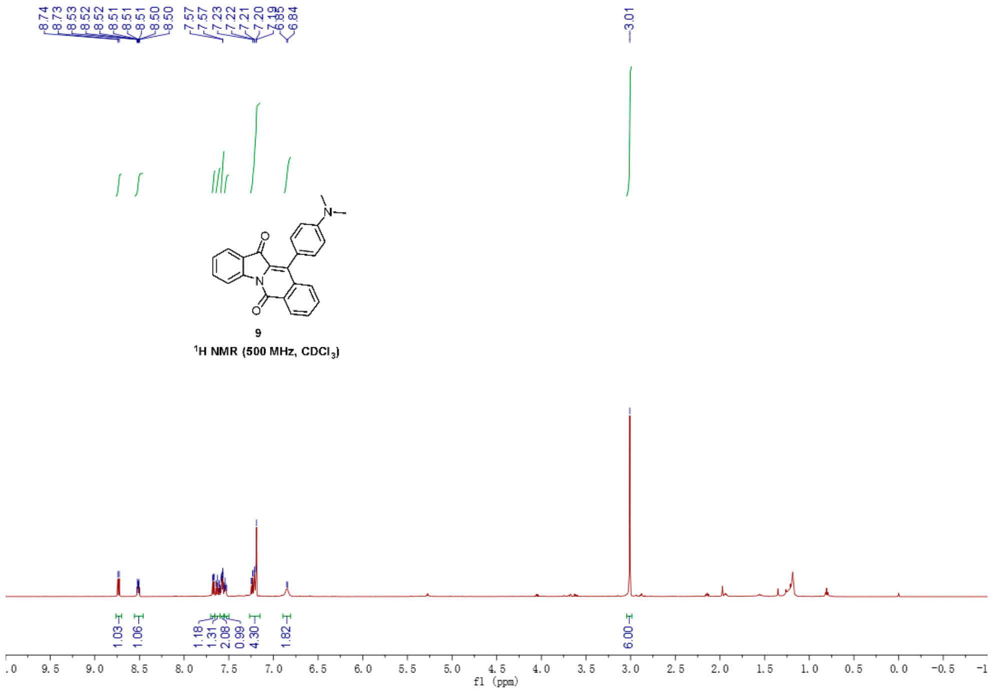


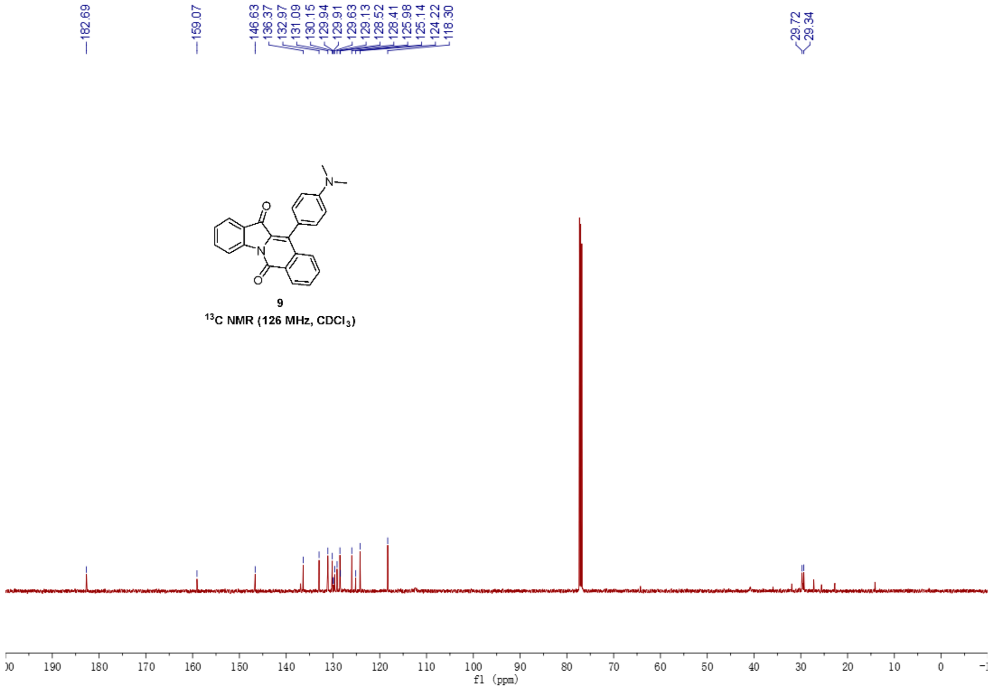


$^{1}$H NMR and $^{13}$C NMR data of compound **9**.

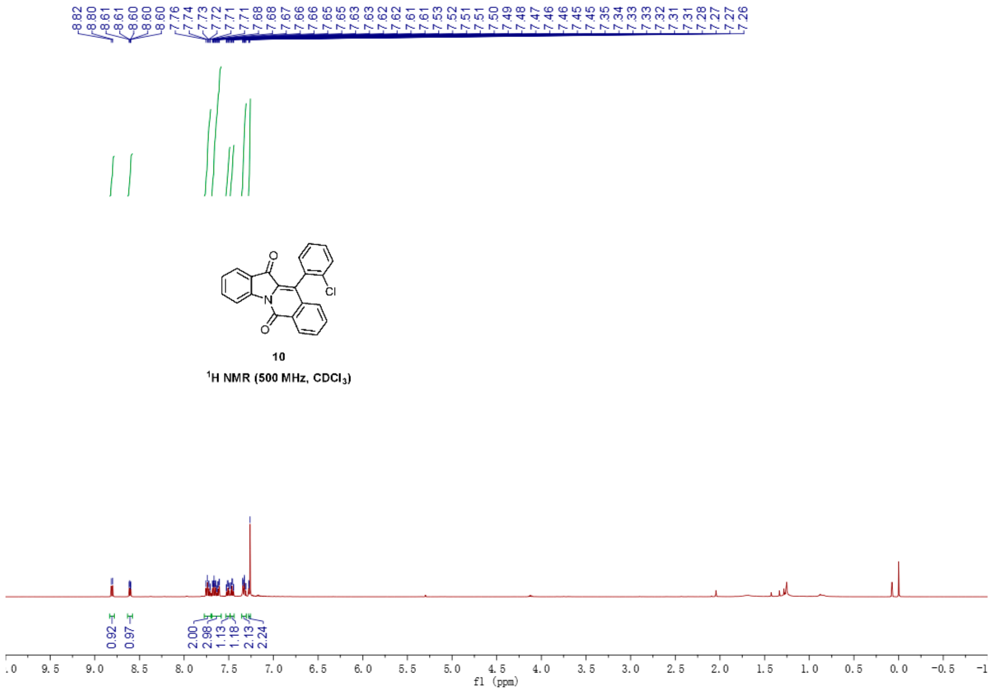


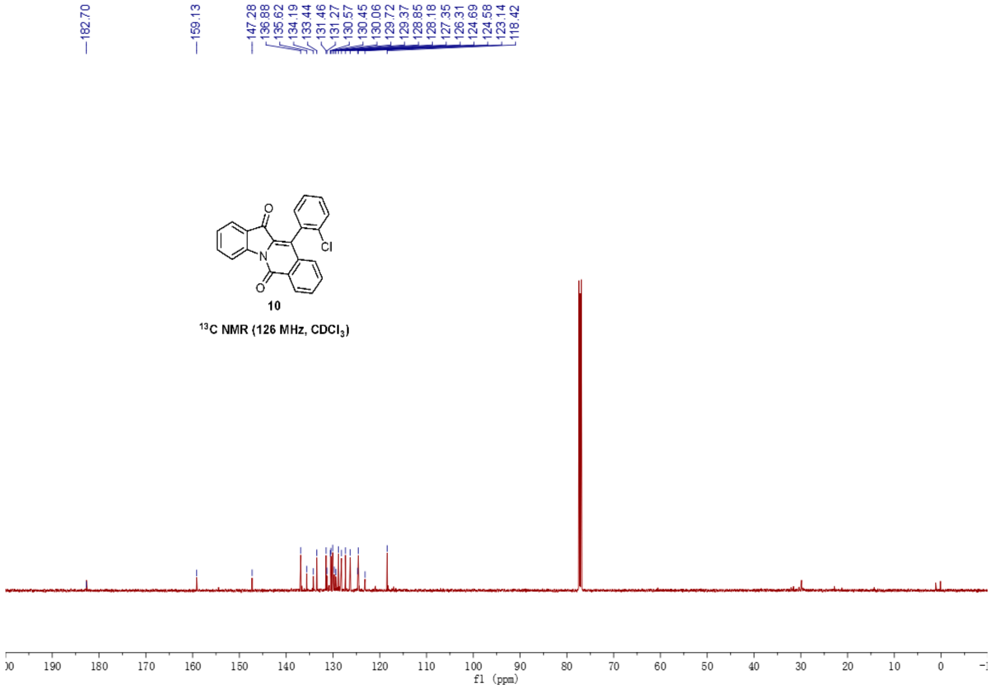


$^{1}$H NMR and $^{13}$C NMR data of compound **10**.

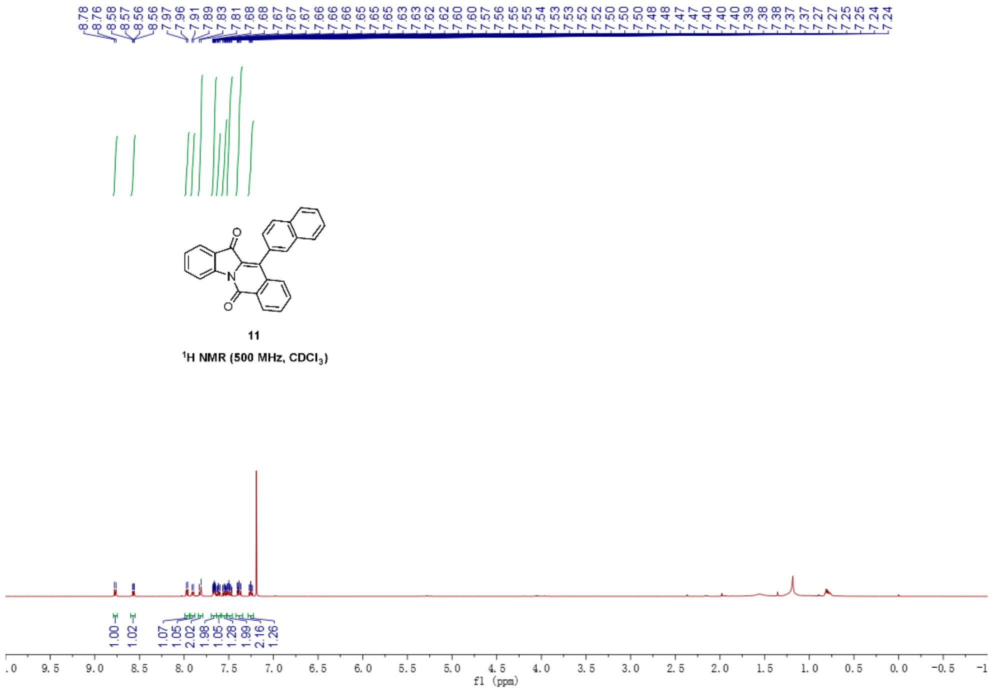

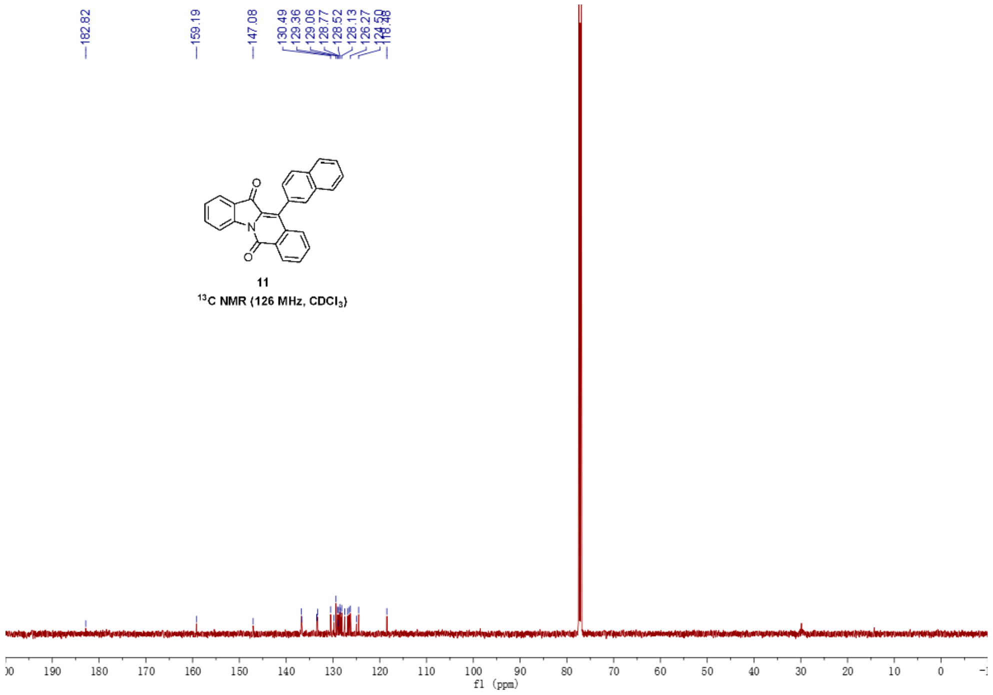

$^{1}H$ NMR and $^{13}C$ NMR data of compound **11**.

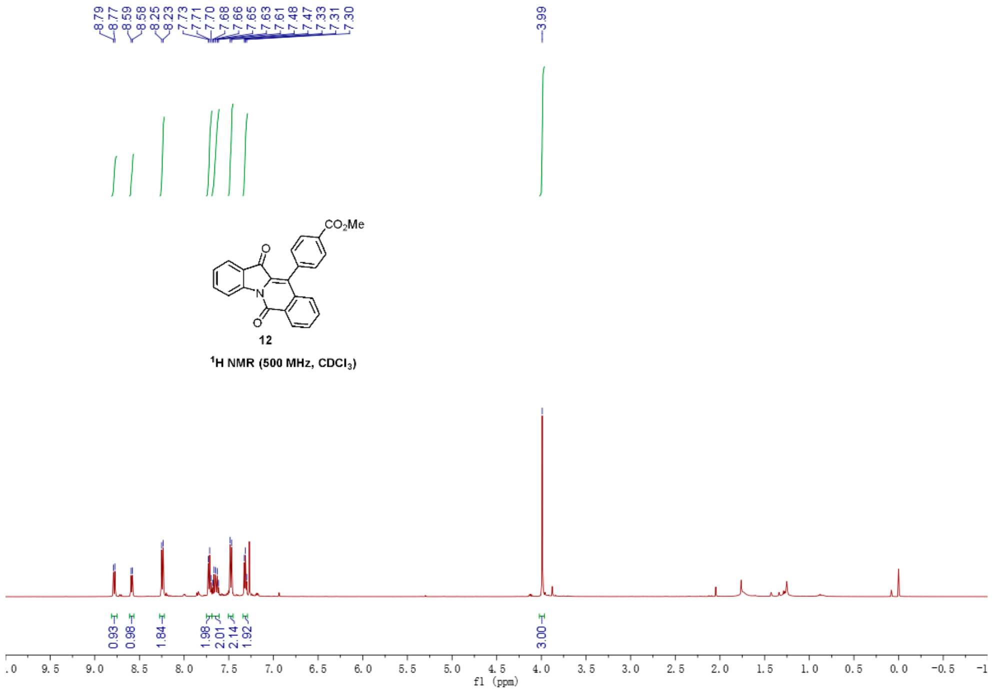

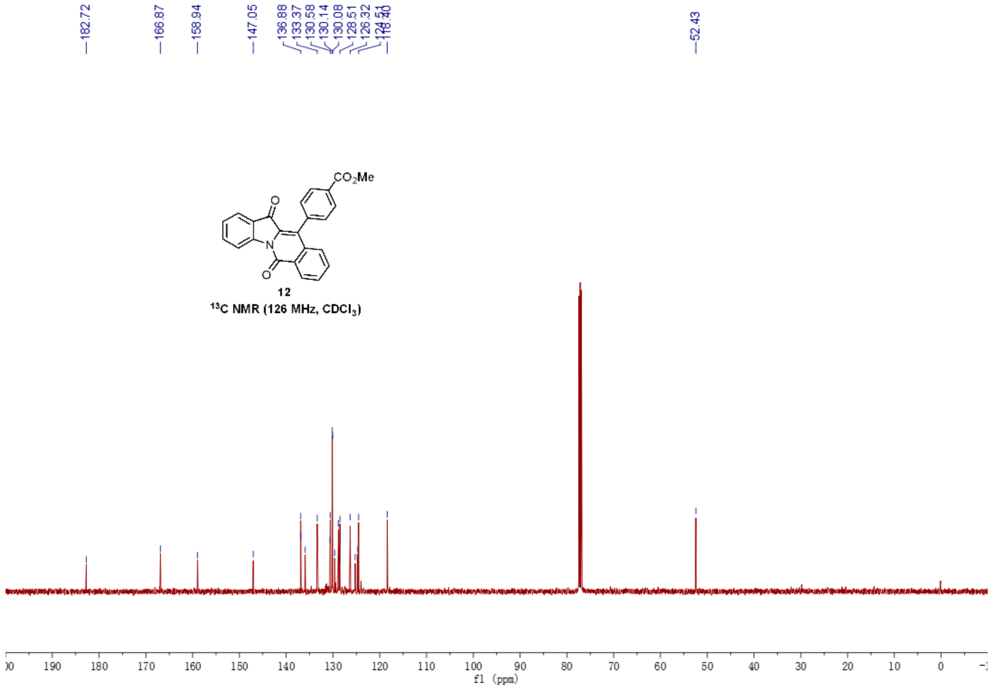

$^{1}$H NMR and $^{13}$C NMR data of compound **12**.

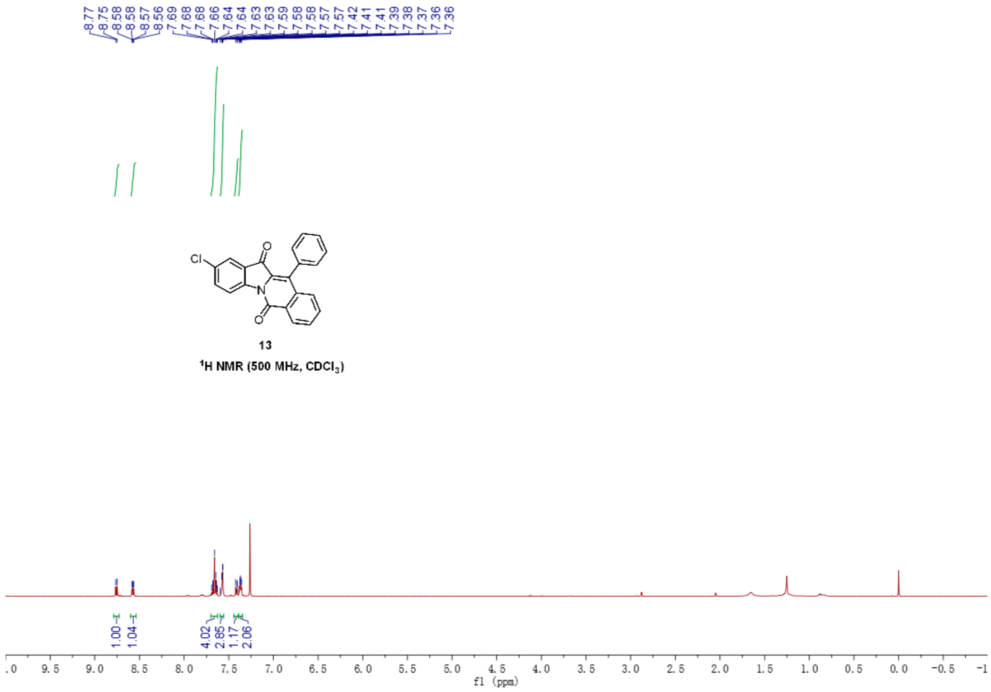


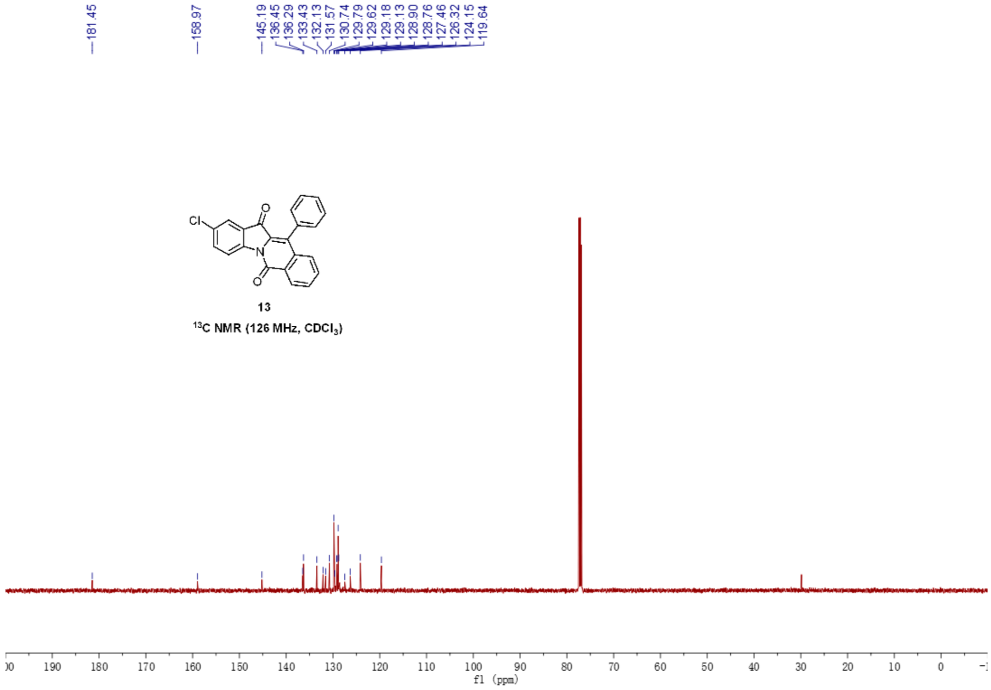


$^1$H NMR and $^{13}$C NMR data of compound **13**.

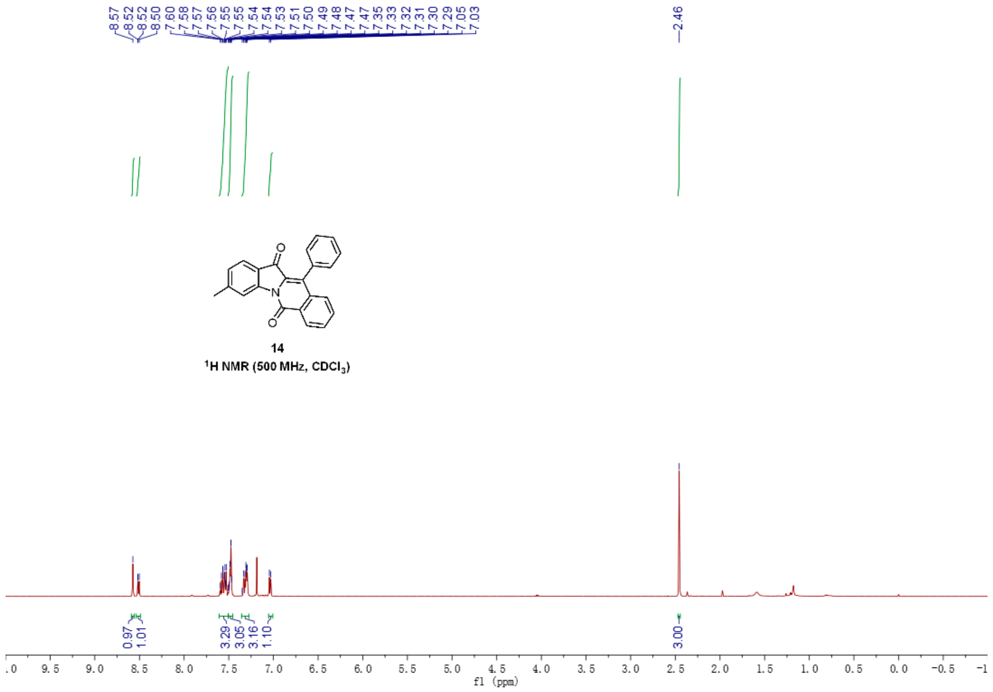


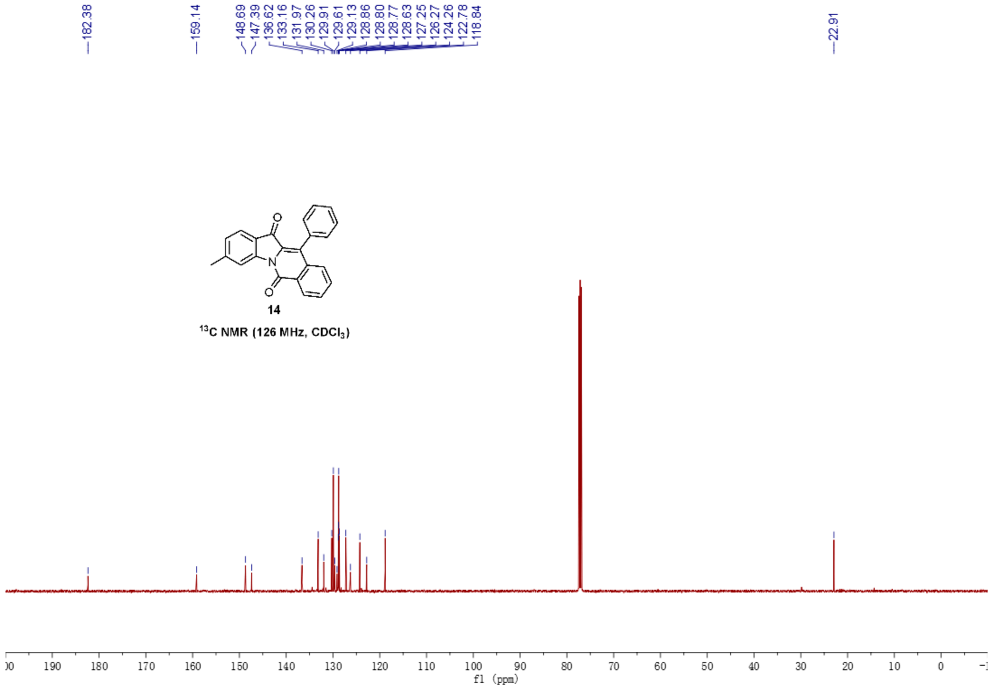


$^{1}$H NMR and $^{13}$C NMR data of compound **14**.

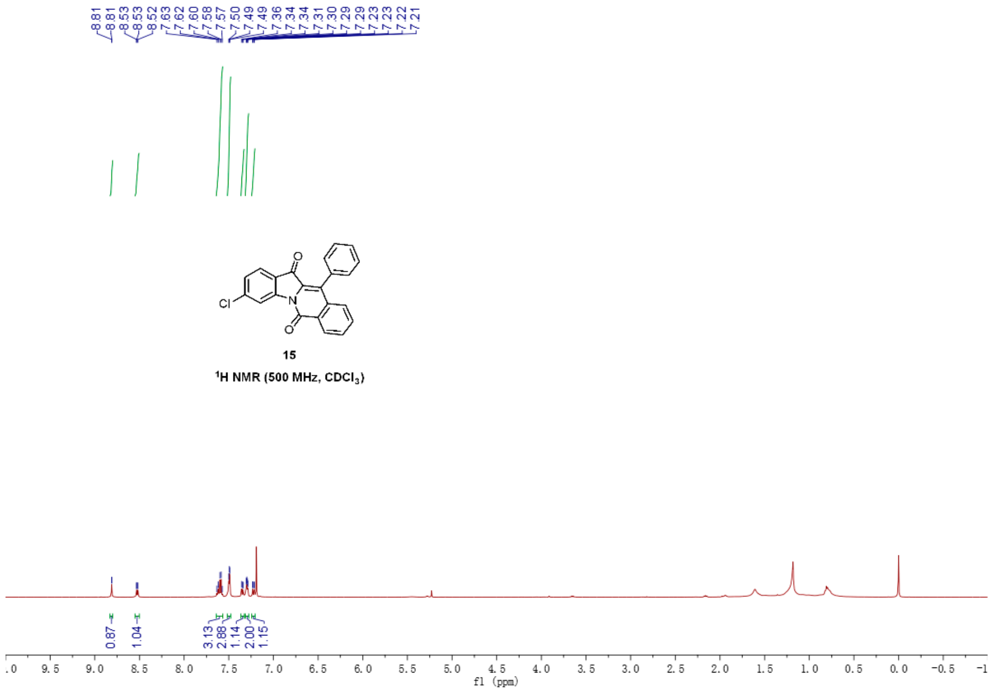


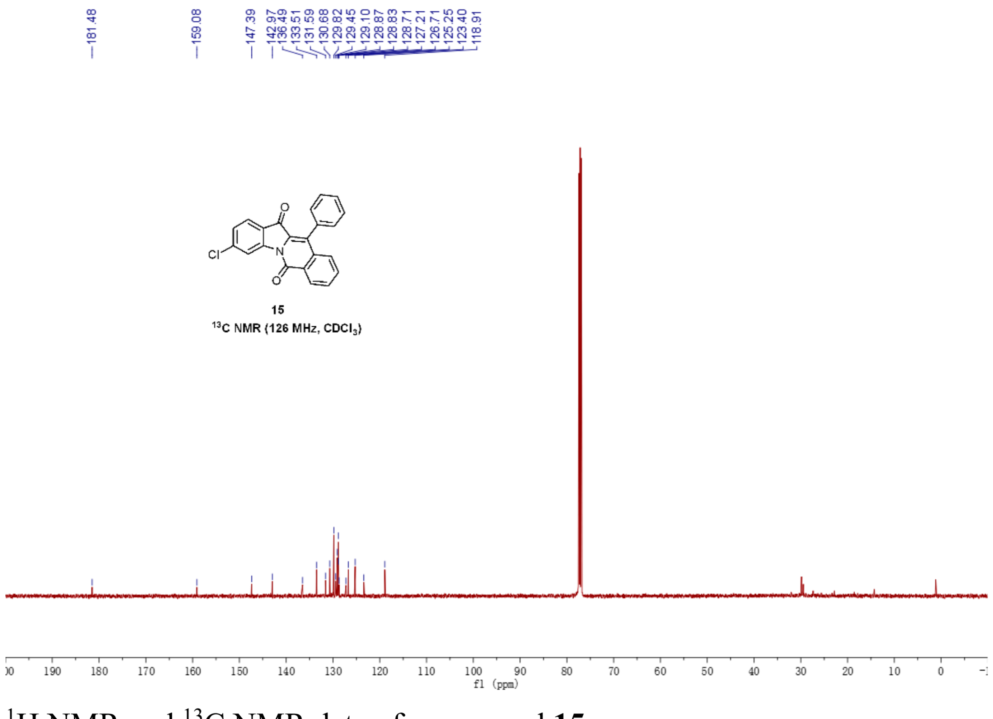


$^{1}$H NMR and $^{13}$C NMR data of compound **15**.

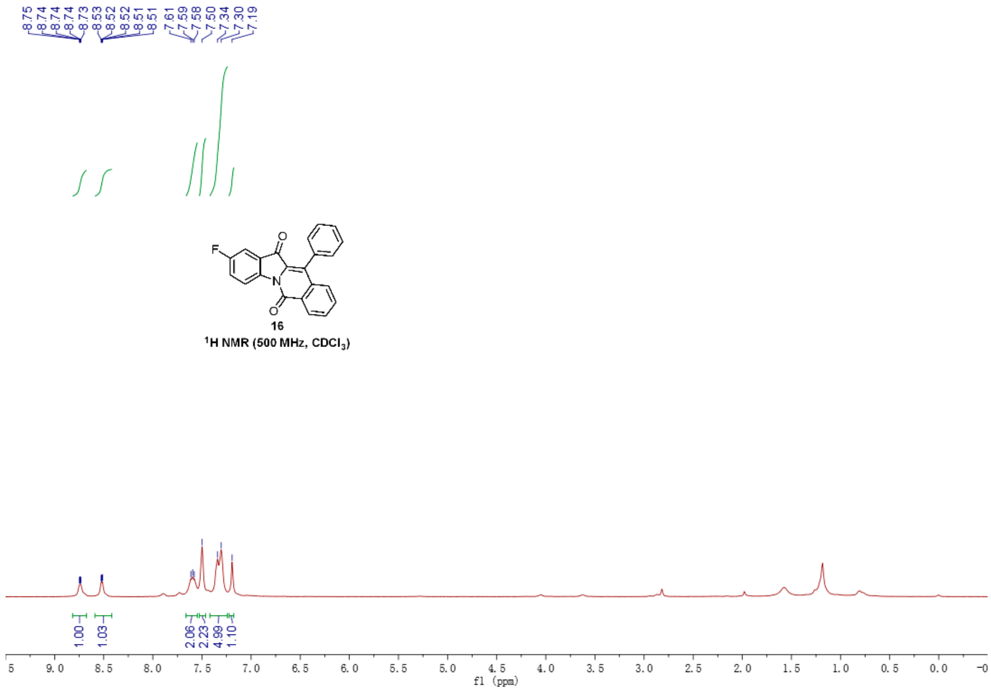


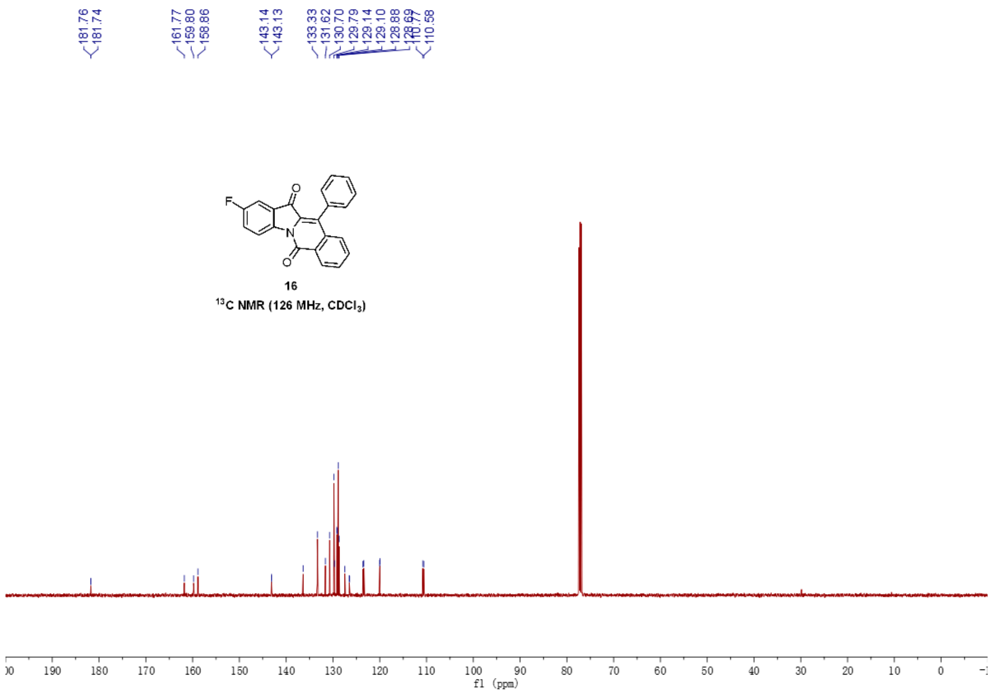


$^{1}$H NMR and $^{13}$C NMR data of compound **16**.

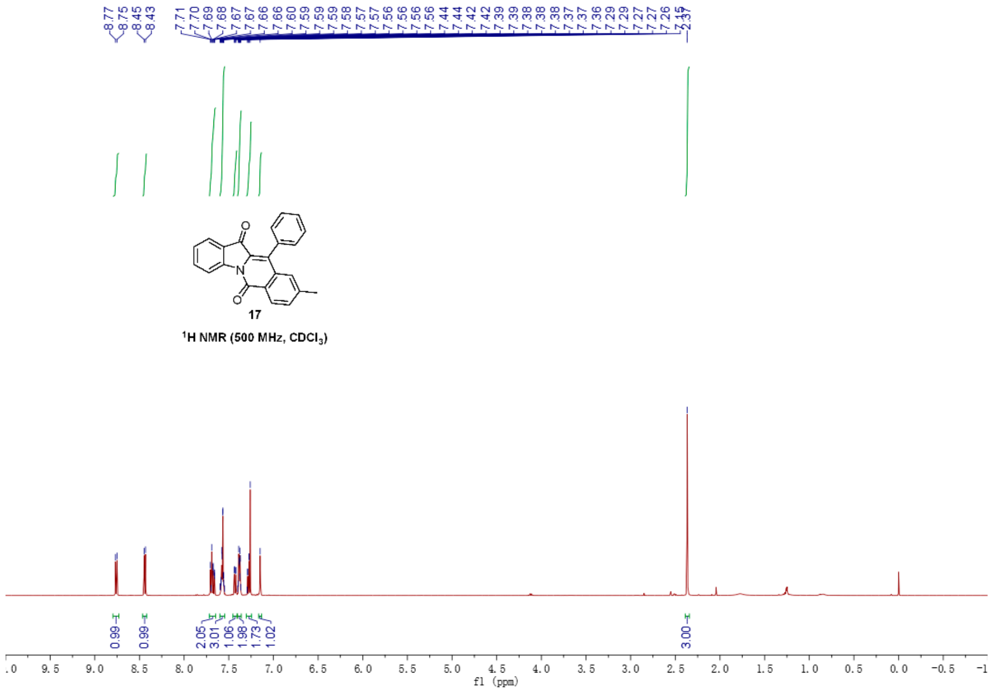

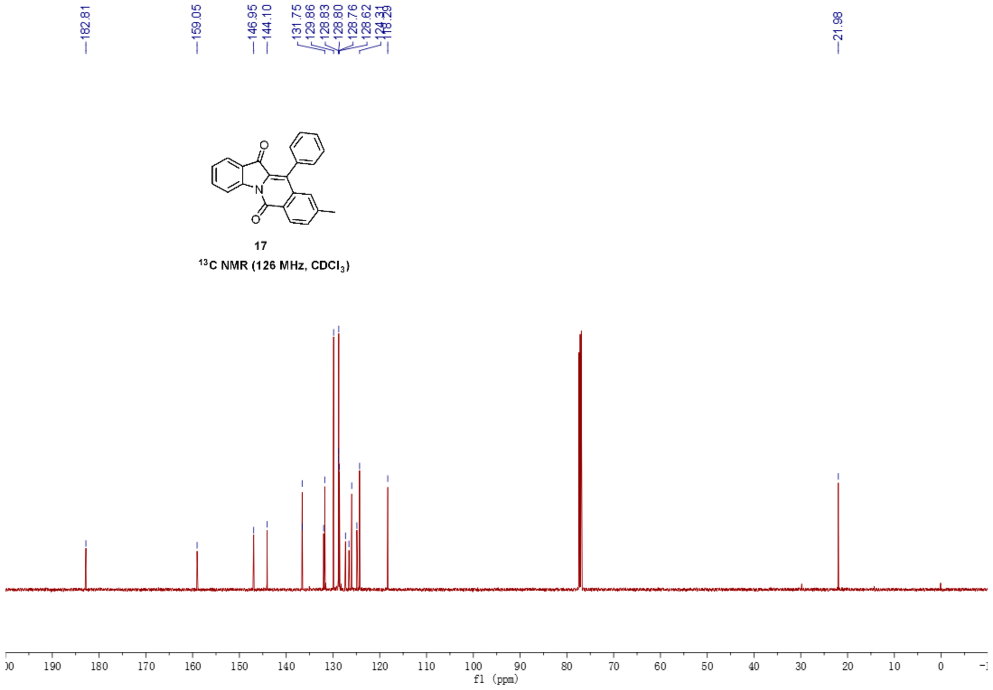

$^{1}$H NMR and $^{13}$C NMR data of compound **17**.

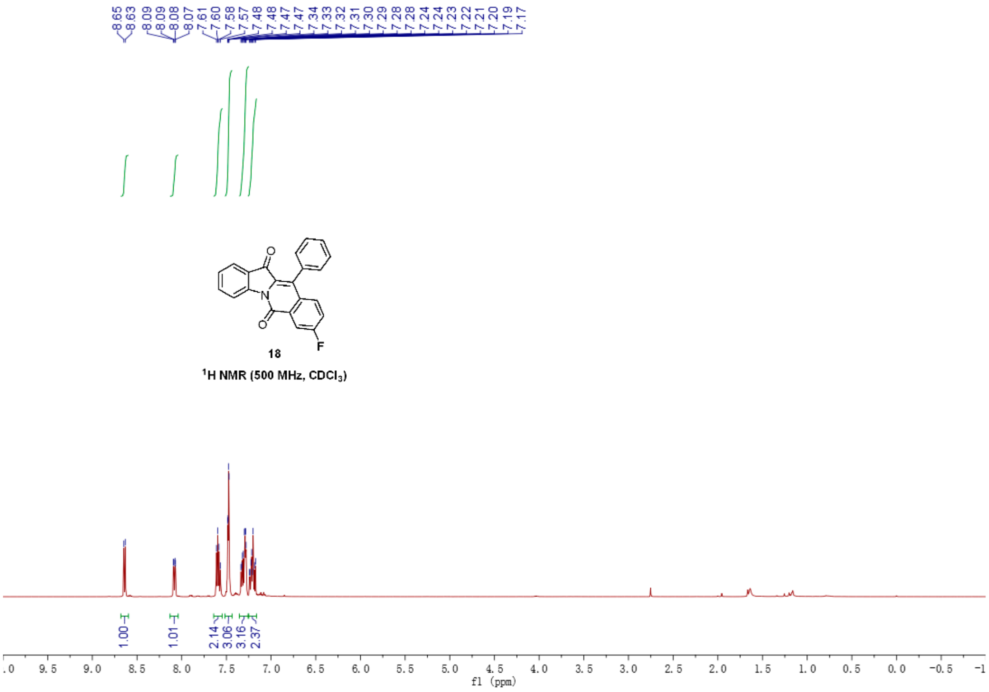


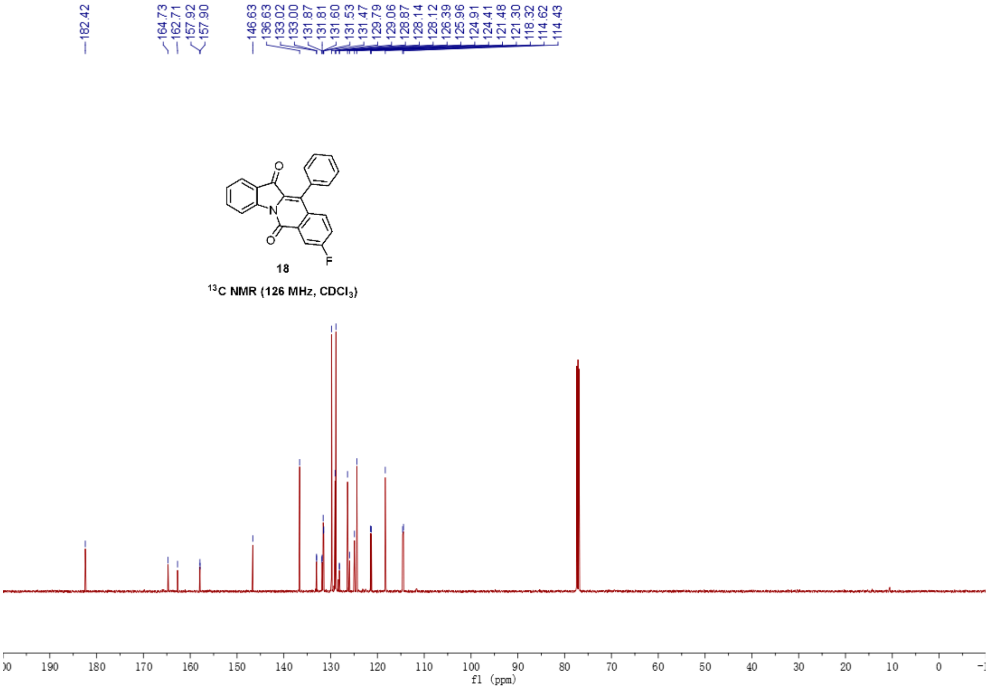


$^{1}$H NMR and $^{13}$C NMR data of compound **18**.

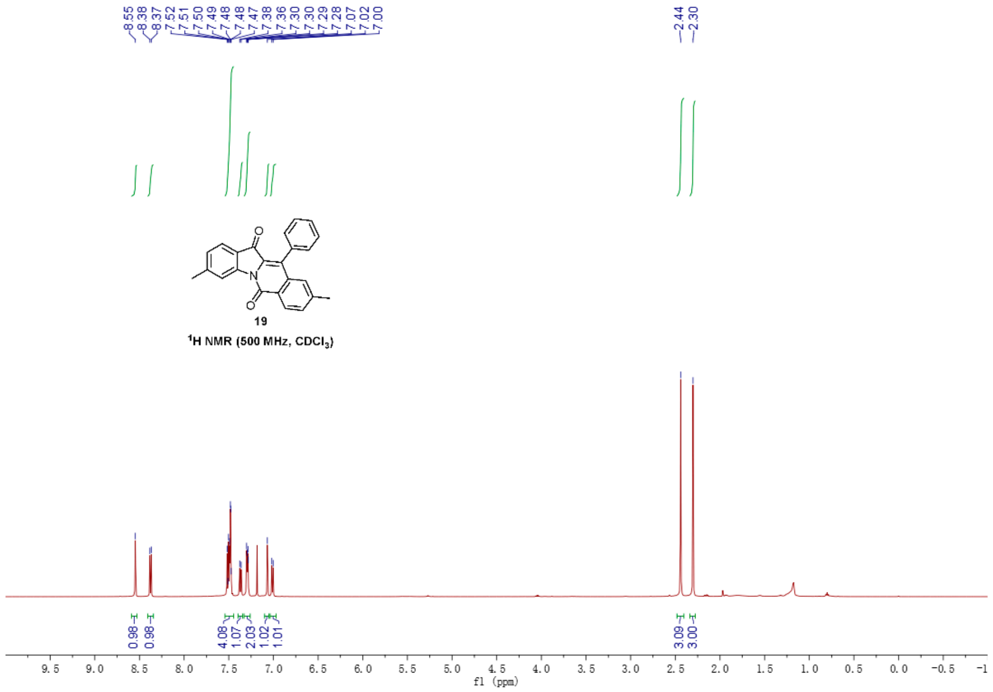


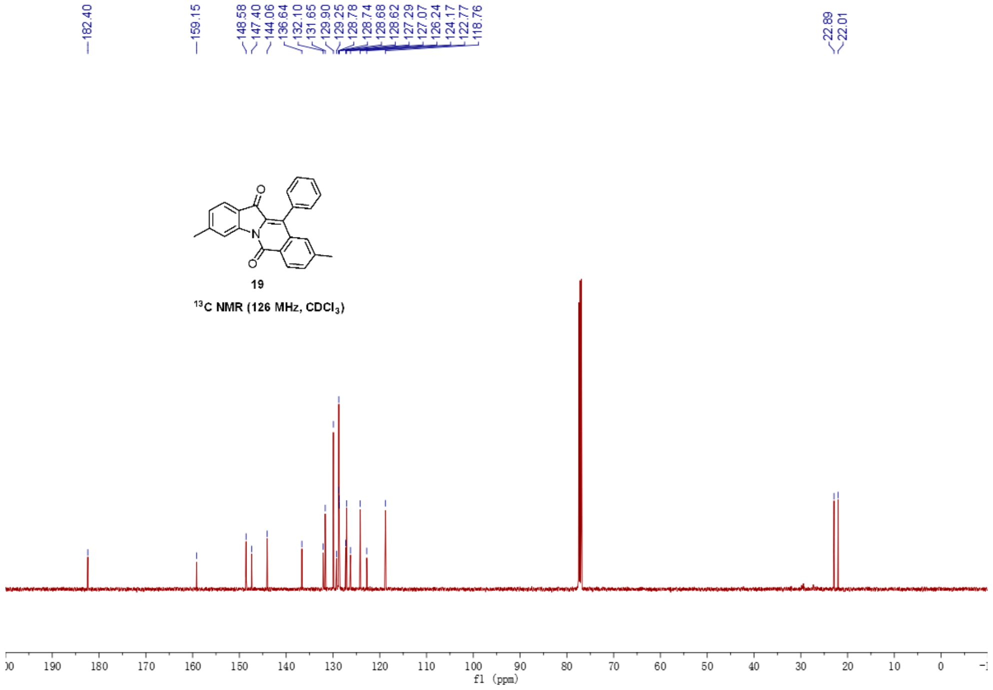


$^1$H NMR and $^{13}$C NMR data of compound **19**.

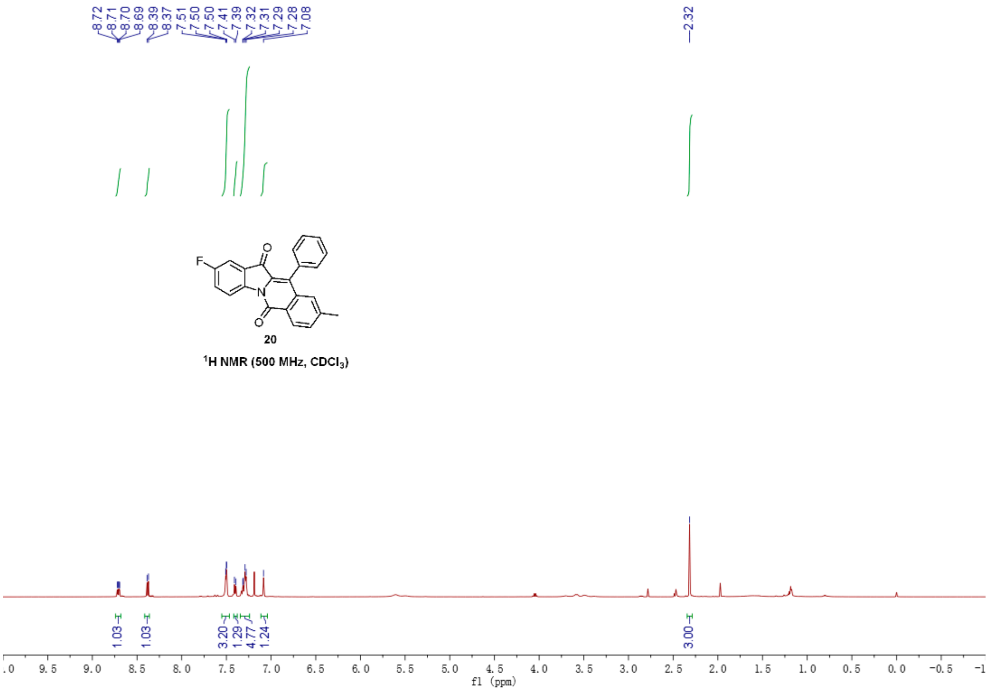


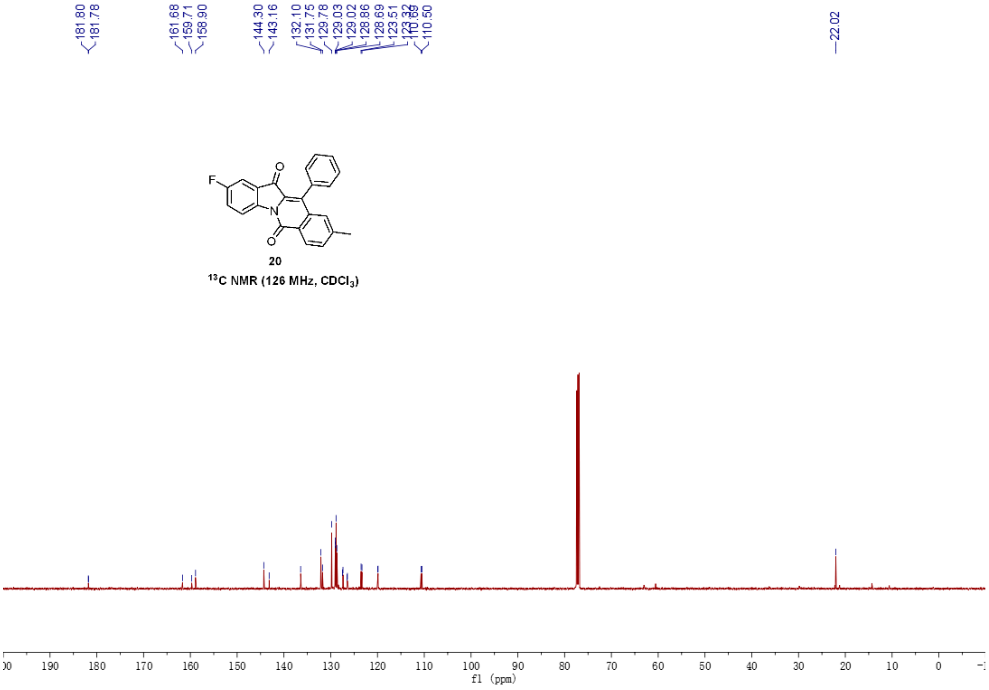


$^{1}H$ NMR and $^{13}C$ NMR data of compound **20**.
